# Age-related Loss of miR-124 Causes Cognitive Deficits *via* Derepressing RyR3 Expression

**DOI:** 10.14336/AD.2022.0204

**Published:** 2022-10-01

**Authors:** Kai Liu, Yongjia Yin, Yuan Le, Wen Ouyang, Aihua Pan, Jufang Huang, Zhongcong Xie, Qubo Zhu, Jianbin Tong

**Affiliations:** ^1^Department of Anesthesiology, Third Xiangya Hospital, Central South University, Changsha, Hunan, China.; ^2^Xiangya School of Pharmaceutical Sciences, Central South University, Changsha, Hunan, China.; ^3^Hunan Province Key Laboratory of Brain Homeostasis, Third Xiangya Hospital, Central South University, Changsha, Hunan, China.; ^4^Department of Anatomy and Neurobiology, Central South University School of Basic Medical Sciences, Changsha, Hunan, China.; ^5^Geriatric Anesthesia Research Unit, Department of Anesthesia, Critical Care and Pain Medicine, Massachusetts General Hospital and Harvard Medical School, Charlestown, MA, USA.; ^6^Postdoctoral Research Station of Department of Anesthesiology, Third Xiangya Hospital, Central South University, Changsha, Hunan, China

**Keywords:** miR-124, ryanodine receptor 3, cognitive aging

## Abstract

Epigenetic alterations of brain contribute to age-related cognitive decline. The challenge now is to identify these tractable epigenetic molecules working as the downstream cell-signaling nodes mediating age-related cognitive decline. Here we reported age-related loss of miR-124 in human and rat brains. To further validate these findings, knockout mice in which one of the three miR-124 genes (miR-124-3) was deleted using CRISPR/Cas9-mediated gene engineering were generated. MiR-124-3 knockout mice developed cognitive deficit phenotype. MiR-124 deficiency in the mouse brain resulted in upregulation of the Ryanodine receptor 3 (*RyR3*) gene, and the cognitive deficits in miR-124-3 knockout mice were ameliorated by knocking down RyR3 expression using RNAi. In addition, miR-124 deficiency facilitated Aβ42-induced neuron apoptosis. Our work suggested that age-related cognitive decline, at least in part, was associated with miR-124 deficiency and subsequently upregulated RyR3 expression in inducing neuronal death.

Age-related cognitive decline (ARCD) is a normal aging deteriorated process and is characterized by the impairment of episodic memory, attention, processing speed, and executive function. It is associated with poor independence and reduced quality of life of elderly individuals [1, 2]. As the aging society is coming worldwide, ARCD becomes a serious public health problem when billions of old individuals lose their own independence due to learning and memory disabilities [3, 4]. Genetic, environmental, and epigenetic factors were considered to contribute to ARCD [5-7]. The molecular pathways that contribute to ARCD have been progressed through various studies using disease like models [8-10]. However, the translational value of those work is limited to apply in tackling ARCD during brain aging.

It is now well documented that neuronal epigenetic alterations contribute to ARCD [11-13]. Small non-coding RNAs (microRNAs, miRNAs) are the key post-translational epigenetic modulation molecules and by binding to messenger RNAs (mRNAs), miRNAs regulate gene expression and subsequently affect cellular phenotype and hence physiological function of individuals. MiR-124 is a “star molecule” modulating the pathogenesis of pathological dementia [14-16]. It is coded by pre-miR-124 at three independent loci (miR-124-1, miR-124-2, and miR-124-3) and is highly expressed in the brain, where its content is 100 times higher than that in other tissues [17, 18]. Moreover, miR-124 expression in the brain is easily affected by chronic stress [19], exercise [20], chronic ischemia [21] and inflammation [22], suggesting that miR-124 may be a common downstream molecule mediating the influence of different environmental factors on brains. However, whether miR-124 modulates ARCD as a key downstream cell-signaling modulator remains unclear.

Here, we tested our hypothesis that miR-124 is a critical epigenetic molecule for ARCD. We reported herein that age-related loss of miR-124 was detected in aged human brains and mouse brains. MiR-124-3 deficiency in knockout mice generated with CRISPR/Cas9 replicated the core phenotypes of normal brain aging. Mechanistically, miR-124 deficiency in the brain led to upregulation of the Ryanodine receptor 3 (RyR3, an intracellular calcium-release channel) and the cognitive deficits in miR-124-3 knockout mice were ameliorated by knocking down RyR3 expression using RNAi. In addition, miR-124 deficiency facilitated β-amyloid-induced neuronal apoptosis.

## MATERIALS AND METHODS

### Brain samples

**Figure 1.**
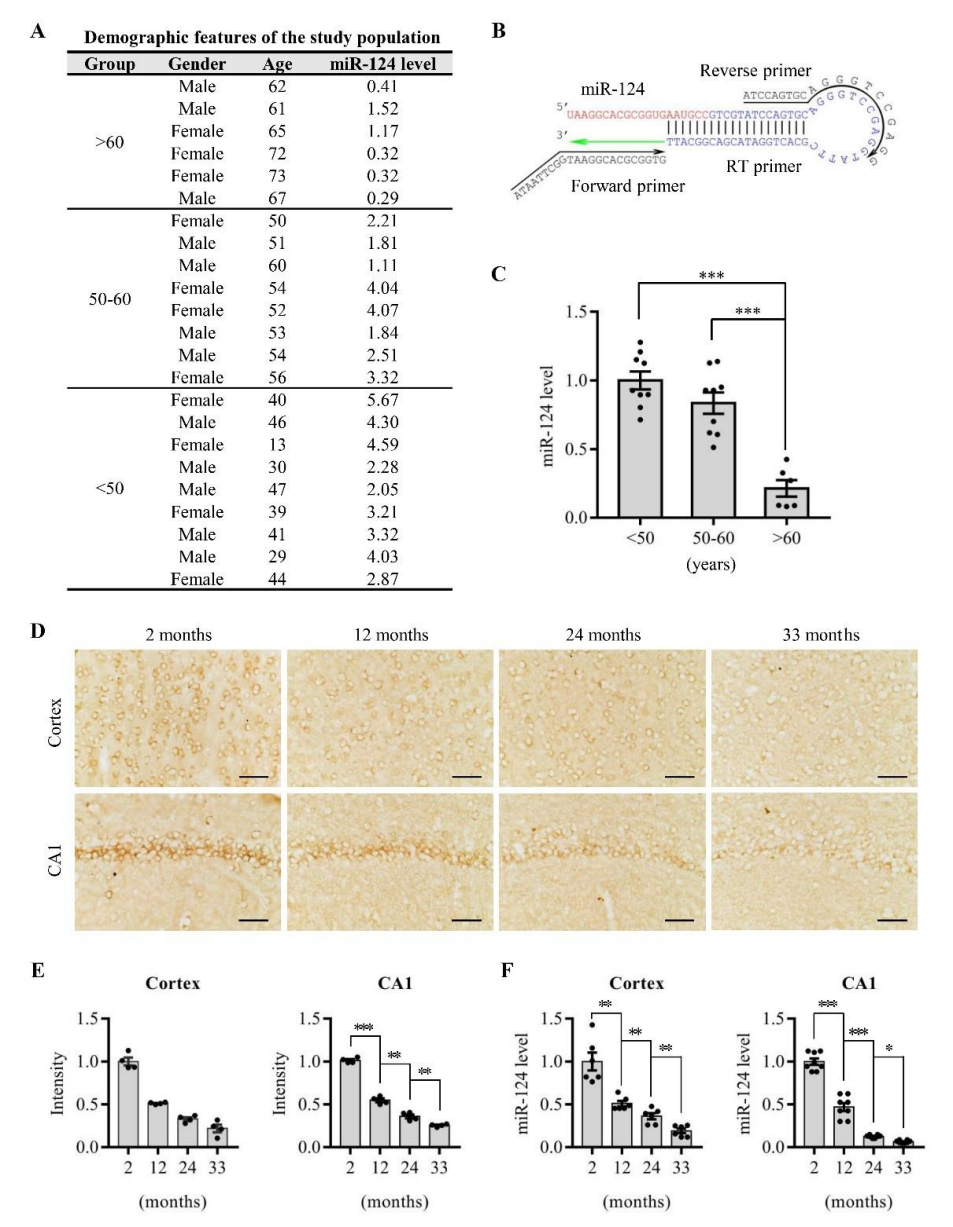
**The expression of miR-124 decreased during aging**. (**A**) Demographic features of 23 Chinese individuals with lateral ventricle ependymoma. MiR-124 level of parietal cortex in operative approach was relative to that of the internal control U6. (**B**) Schematic diagram of targeted sequence of miR-124 PCR primer sets. (**C**) qPCR analysis of miR-124 of parietal cortex in individuals; Human U6 was used as internal control (mean ± SEM; n=6, 8 and 9 samples for >60, 50-60 and <50 group, respectively; ***P<0.001; one-way ANOVA followed by Tukey *post hoc* Test). (**D**) Representative images and corresponding optical density statistics (E) of miR-124 *in situ* hybridization staining in the hippocampal CA1 area and the parietal cortex of rats of different ages; (Scale bar=40 μm; mean ± SEM; n=4 rats per group; *P < 0.05, **P < 0.01, ***P<0.001; one-way ANOVA followed by Tukey *post hoc* Test). (**F**) qPCR analysis of miR-124 of the parietal cortex and the hippocampal CA1 area of rats of different ages. U6 was used as internal control (mean ± SEM; n=6 rats for cortex and 8 rats for hippocampus per group; *P < 0.05, **P < 0.01, ***P<0.001; one-way ANOVA followed by Tukey *post hoc* Test).

The Institutional Review Board of the Third Xiangya Hospital approved the use of the human brain tissue (www.chictr.org.cn/listbycreater.aspx.ChiCTR1900028072). Each patient provided Written informed consent for medical or scientific research use of excised brain tissue from 23 patients (Fig. 1A) whose parietal cortex tissues were collected with lateral ventricle ependymoma via an operative approach with a sterile fine needle during surgery.

### Animals

Rats and mice (young and old) were provided free access to water and food in their home cages. The lights were maintained on a 12-h light/dark photoperiod. Experiments were performed according to the National Institutes of Health Guide for the Care and Use of Laboratory Animals. The animal care and use committee of Central South University approved the experimental protocols.

### miR-124-3 knockout mice

The SP6 promoter-driven nuclear-targeted humanized Cas9-encoding mRNA (25 ng/μl) and the T7 promoter-driven production of a customizable synthetic sgRNA followed by tracrRNA-derived sequences at the 3' end (12.5 ng/μl) were transcribed and then microinjected into the cytoplasm of one-cell-stage C57BL/6 mouse embryos. The embryos were then implanted into surrogate mother mice. To achieve germ line transmission of the miR-124-3-null allele, the founder mice were backcrossed with C57BL/6 mice. Primer pairs for the wild-type and knockout alleles were designed (Supplementary Table 1). Genomic DNA was extracted from mouse tail clips using the Mouse Tail SuperDirect PCR Kit (Foregene, Beijing, China) according to the manufacturer’s instructions to distinguish those mice of wild-type, heterozygous, and homozygous phenotypes, respectively. Genotyping was performed using PCR assay and a T-Gradient PCR instrument (Biometra, Germany).

### Behavioral tests

#### DMP water maze task

The DMP water maze task was slightly modified from the protocol used by *Steele and Morris*[23]. Briefly, all mice were pre-trained using four trials per day for 5 days. Each mouse was released at an arbitrary quadrant and facing the side walls. The hidden platform was located at five separate places within the pool. A trial ended after the mouse spent 30 seconds on the platform. During pre-training, we only measured the escape latencies between trials 1 and 2. After pre-training, the mice were given the testing tasks using memory intervals (inter-trial intervals between trials 1 and 2) of 5 sec, 20 min, 2 h, or 4 h (not including a 30-sec period spent on the platform). Each testing phase period was 3 days. Testing phase performance was calculated by subtracting the trial 2 time /path-length for each mouse from its trial 1 time/path-length. Greater time/path-length differences indicated better performance.

#### Morris water maze standard hidden platform task

The Morris water maze test was performed and analyzed to measure memory function. Three times per day for 4 days, each mouse was placed in a pool containing a submerged platform located at a fixed position. Latency and speed were recorded using a SMART digital tracking system (SMART JUNIOR v1.0). During the probe test, the platform was removed from the pool and the mouse was allowed to swim freely for 60seconds. The number of platform-site crossovers, percentage of time spent in the target quadrant, and time to the first target-site crossover were analyzed.

#### Footprint analysis

The plantar surfaces of the hindlimbs and forelimbs were painted with red and blue inks and the mouse was allowed to run on white paper to obtain a footprint record. Stride length and width were recorded to evaluate motor function [24].

#### Rotarod test

A rotarod apparatus with a 3-cm diameter rod was used to assess balance and motor coordination [25]. On the training day, each mouse was trained on the rotarod at a speed of 4 rpm for 300sec. On the testing day, each mouse was tested at a speed of 20 rpm, and the time before falling was recorded.

#### Novel object recognition

The novel object recognition experiments were used to evaluate memory [26]. Twenty-four hours after habituation, each mouse was allowed to explore (10 min) an arena with two identical objects placed at an equal distance. The next day, the mouse was placed in the arena, but one of the objects was replaced with a novel object. The mouse was again allowed to explore for 10 min. The time spent exploring each object was recorded and the discrimination index (DI) was calculated using the formula DI = TN/(TN+TF), where TN = time spent exploring the novel object and TF = time spent exploring of familiar object.

#### Forced swimming test (FST)

The FST is a valid test for interpreting, assessing, and determining susceptibility to negative moods (e.g., depression) [27]. Each mouse was allowed to swim freely for 6 min. Immobility time during the last four minutes was recorded.

### Histology and immunostaining

Brain tissue was fixed in 4% paraformaldehyde. It was then dehydrated using sucrose, embedded with OCT, and finally cut into 20-μm sections using a cryostat. For immunohistochemistry, the sections were treated with 3% H_2_O_2_ and blocked with 5% BSA in 0.01 M PBS plus 0.1% Triton X-100. After incubation in primary antibody (anti-Iba1, 1:1,000, cat#: 019-19741, Wako, Japan) at 4°C overnight, the sections were incubated with secondary antibodies (1:200, Cat#: BA-1000-1.5, Vector Labs, Olean, NY, USA) and then incubated in ABC solution (1:100, Vector company). Diaminobenzidine (Vector) and counterstaining with hematoxylin to display nuclei were used to visualize the sections. Primary antibodies were controlled by isotype IgG (CST, cat#: 2975S, USA) to validate antibody specificity. Based on the Iba1 staining, the resting and activated microglia in the CA1 and DG were distinguished following the method previously established by Cerbai F *et al.* [28]. For the Nissl staining, one set of hippocampus sections was randomly selected, mounted on gelatin-coated slides, and dried. Crystal violet was used to stain the sections for cell counting. Images were taken in five sections (at -1.22, -1.46, -1.58, -1.82, and -2.06 mm relative to the bregma; all same magnification) in the CA1 region. The FD Rapid Golgi Stain Kit was used for the Golgi staining, according to the manufacturer's instructions[29]. Briefly, brain sections of 100-μm thickness were mounted on gelatin-coated slides and then dried. The sections were then stained, dehydrated in graded ethanol, cleared in xylene, and covered with a coverslip. Four to five apical dendrites (tertiary or quaternary) and three to five basal dendrites (secondary or tertiary) with at least one branch point were selected from each neuron for counting. The visible spines along the branch segment (10 μm in length) were counted.

### Viral vectors and stereotactic injection

For miR-124-3 overexpression and RyR3 knockdown, virus was constructed at the Genomeditech company (Shanghai, China). Partial mmu-mir-124-3pri-microRNA sequences cloned with primer pairs (forward: 5'GCTCTGCACCCGTCAGAAGACTG3', reverse: 5'C CCTTTGTCTTCTGGCCCTCG3') were inserted into the CMV promoter of lentivirus infectious virions, pGLMV-MA1. ShRNA construct was designed to target the specific sequence in mmu-RyR3 (5'GAACATA TGCCCAACGATT3') [30] and inserted into the CMV promoter of adeno-associated virus pAAV-mCherry-ShRNA. For miR-124 mimics (Sangon Biotech, China) injection, implantation was conducted 7 days prior to the electrophysiological analysis. The miR-124 mimics (1 ng in 0.5 μl aCSF for each mouse) or control microRNA (1 ng in 0.5 μl aCSF for each mouse) was mixed with transfection reagent (0.1 μl mixed with 0.4 μl aCSF) (Lipofectamine 2000; Invitrogen, Paisley, UK) [31]. Each mouse was anesthetized using isoflurane. The head of the mouse was then placed in a stereotactic apparatus (RWD, China). The skull was exposed, and a small craniotomy was performed. The mice were bilaterally microinjected in each hemisphere following specific coordinates that were relative to the bregma (anteroposterior (AP), -2.18 mm; mediolateral (ML), ±2.00 mm; dorsoventral (DV), -2.10 to -1.60 mm). A total of 0.5μl was injected into two hemispheres. A 10-μl syringe (Hamilton, USA) with a 33-gauge metal needle (Hamilton, USA) was used for all microinjections. When each injection was complete, the needle was kept in place for 10 min to allow the viral vector to diffuse away from the needle track. It was then slowly withdrawn.

### Western blot

Total proteins from tissues and cells were extracted in RIPA buffer (Beyotime, Jiangsu, China) containing protease inhibitor cocktail (Sigma, St. Louis, USA). To isolate the protein on the membrane, ultracentrifugation was applied to isolate cellular membrane after the tissues mashed mechanically. Then proteins were extracted with RIPA buffer from the precipitation. The total protein was separated using 10% SDS-polyacrylamide gel electrophoresis and then transferred to PVDF or NC membranes. After blocking with 5% milk, the proteins were detected using specific primary antibodies (Supplementary Table 4). The fluorescigenic secondary antibodies (1:5,000, LI-COR, cat#: 926-32211(goat anti-rabbit) and 926-68070(goat anti-mouse), USA) were then applied. All primary antibodies were controlled by isotype IgG (CST, cat#: 2975S, USA, Proteintech, cat#: 66360-3-Ig, China) to validate antibody specificity and only specific band of the antibody showed at certified molecular weight site was accepted. All antibodies were diluted with antibody dilution buffer (Beyotime, China) following the recommendations in the reagent supplies manual.

### Enhanced in situ hybridization

Mature miR-124 was detected using a double-DIG-labeled miRCURY LNATM miRNA detection probe has-miR-124-3p (sequence/5DiGN/GGCATTCACCG CGTGCCTTA/3DiG-N/) (Qiagen, Germany) and scramble probe as sequence/5DiGN/ACACTCG/ IXNA_ C/ggC TTATTgCCg/3DiG-N (Sangon Biotech, China). The enhanced *in situ* hybridization procedure was performed according to the Enhanced Sensitive ISH Detection Kit I (Boster, Cat#: MK1030, China) protocol. Briefly, the paraformaldehyde-fixed frozen samples were cut into 20-μm thick sections and mounted on amino silane coated slides (Solarbio, China). Each slide was then washed with methyl alcohol with 0.3% hydrogen peroxide (30 min, room temperature). After incubation with pepsin (2 min, 37°C) and pre-hybridization (3h, 60°C), slides were hybridized with miRNA probe (20 nM, 60°C) overnight After a stringent washing, all sections were blocked using blocking solution (60 min, 37°C). Each section was then sequentially incubated with biotinylated mouse anti-DIG antibody (60 min, 37°C), streptavidin-biotin complex (60 min, room temperature), and biotinylated peroxidase (60 min, room temperature), with a 20-min wash in 0.5M PBS between each step in the sequence. Following staining with 3, 3-diaminobenzidine, the slides were dehydrated in a graded ethanol, cleared in xylene, and covered with coverslips, then coated with neutral resins.

### Electrophysiological recording and stimulation

The mice were decapitated, and the brains were immediately immersed in ice-cold artificial cerebral spinal fluid (aCSF; 2.0mMKCl, 12mM MgSO_4_, 0.2mM CaCl_2_, 1.3mM NaH_2_PO_4_, 10mM glucose, 220 mM sucrose, 26mM NaHCO_3_) to cut at 300 μm by a Vibro slice microtome (VT 1200S, Leica, Germany). Slices were incubated (30 min,34°C) and then kept at room temperature (25±1°C) for an additional 2-8 h in recording aCSF (126 mMNaCl, 2.5mMKCl, 1.25mM NaH_2_PO42.0mM CaCl_2_ 1.0mM MgSO_4_ 26mM NaHCO_3_ and 10mM glucose). All solutions (slicing and recording aCSF) were saturated with 95% O_2_/5% CO_2_ (v/v). For field excitatory postsynaptic potentials (fEPSPs) recording, the slices were placed in a recording chamber superfused (3 ml/min) with recording aCSF (32-34°C) and recorded in current-clamp mode using an Axon MultiClamp 700B amplifier (Molecular Devices, USA) with recording aCSF-filled glass pipettes (2-5 MΩ). The test stimuli consisted of monophasic 100-μs pulses (0.033 Hz) at a constant current (intensity adjusted to produce 25% of the maximum response). The synaptic transmission strength was determined via measurement of the initial (10-60% rising phase) fEPSP slopes. LTP was induced using one train of high-frequency stimulation (test stimulus intensity, delivered at 100 Hz and lasted for 1s). To induce LTD, one train of low frequency stimulation (1 Hz, 100 s) was applied. The magnitudes of LTP and LTD were calculated on basis of the averaged EPSC values during the last 30 min of the LTP and LTD summary graphs. All electrophysiology experiments were performed and analyzed under blinded conditions.

### Calcium imaging

The preparation of brain slices for calcium imaging was the same as that of the electrophysiological studies described above, except that the slice thickness was 400 μm. Imaging process was mainly referred to other reports [32, 33]. Briefly, after recovery in the recovery aCSF (30 min, 34°C), the brain slices were transferred to the oxygenated recording aCSF containing 2 μM Fluo-4, AM (Invitrogen) and 0.1% Pluronic F-127 (Invitrogen) for 30 min. Individual slices were transferred to a recording chamber with Mg^2+^-free aCSF. The confocal fluorescence images (488-nm wavelength) were acquired using the 4×, 10×, or 20× objectives on a Nikon Ti microscope. The images were analyzed using ImageJ software (NIH). The red fluorescence (mCherry fluorescent protein) was detected at a 561-nm wavelength. The images were merged to confirm that the calcium imaging was focused on the neurons.

### RT-PCR

Trigol (Dingguo, Co.) reagent was used to extract total RNA from tissues and cells. The primers were designed to overlap the exon junction areas. A mirVana miRNA isolation kit (Ambion, Cat#: 4458237, Austin, TX, USA) was used to extract the miRNAs. The YRBIO miRNA qPCR Detection primer sets, and the U6 snRNA PCR primer (Yingrun Biotechnology, China) are presented in Supplementary Table 1. M-MLV Reverse Transcriptase (Invitrogen, San Diego, CA, USA) was used for the first-strand cDNA synthesis.

### Luciferase reporter assay

The partial pre-miR124-3 sequence was inserted into the pRNA-CMV3.1 vector and the 3’-UTR sequence including the seed sequence was inserted into the pGL3-Promoter vector (Promega, Madison, WI, USA). For the luciferase analysis, HEK293 cells were co-transfected with different concentrations of pre-miR124-3 plasmid, 100ng target report plasmid, and 40ng pRL-CMV-Renilla plasmid. Mutated 3’-UTR and empty vector were used as controls. The luciferase reporter assays (Promega) were performed 48 hours after transfection, and the luciferase activity was quantified using a GloMax 20/20 Luminometer (Promega). The ratios of the firefly to Renilla luciferase activities were used to calculate relative luciferase activity.

### Neuronal apoptosis analysis

Three days after Aβ1-42 (0.5 μl/ 0.5mM) injection in the hippocampus (AP, -2.00 mm; ML, ±1.30 mm; DV, -2.10 to -1.60 mm), the mice were humanely euthanized, and the brains were cut into 20-μm sections. Sections with obvious needle track were mounted on amino silane coated slides (Solarbio, China). Neuronal apoptosis was analyzed using *In Situ* Cell Apoptosis Detection Kit V (POD) (Boster, China) testing protocol. Briefly, the sections were incubated with DIG-labeled UTP and terminal nucleic acid transferase (2 h, 37°C). After incubation with blocking solution (60 min), the slides were sequentially incubated with biotinylated mouse anti-DIG antibody (60 min, room temperature) and streptavidin-biotin-peroxidase complex (60 min, room temperature). Positive cells were selected as 3, 3-diaminobenzidine-stained and were counted and compared between wild-type and knockout mice.

### Statistical analysis

All results were presented as mean ± SEM values. Prism software (GraphPad Software, Inc) was used to perform the statistical analyses. When two groups were compared, the *t* test (passed normality test) or the non-parametric Mann-Whitney *U* test (did not passed normality test or n<6) was applied. A one-way ANOVA or two-way ANOVA with repeated measures was performed when multiple groups were compared. Differences were considered statistically significant at *P ≤ 0.05, **P ≤ 0.01, and ***P ≤ 0.001. Sample sizes were determined based on experience. Detailed statistical information for all study results is presented in Supplementary Table 2.

## RESULTS

### MiR-124 was downregulated in the aged subjects

To detect changes of miR-124 levels during brain aging, we examined miR-124 levels in parietal cortex in operative approach of 23 Chinese individuals with lateral ventricle ependymoma (Fig. 1A). Compared with the adults younger than 60 years, miR-124 levels decreased significantly in individuals older than 60 years (Fig. 1C). To confirm the finding of age-related changes in miR-124, we also examined miR-124 levels in the brains of rats of different ages using *in situ* hybridization and RT-PCR (Fig. 1D, E and F). In both the hippocampus and the parietal cortex tissues of rats, miR-124 levels gradually decreased during aging (Fig. 1D, E and F). Taken together, these results indicated that miR-124 was downregulated in aged subjects.

### MiR-124 deficiency was reproduced in miR-124-3 knockout mice

**Figure 2.**
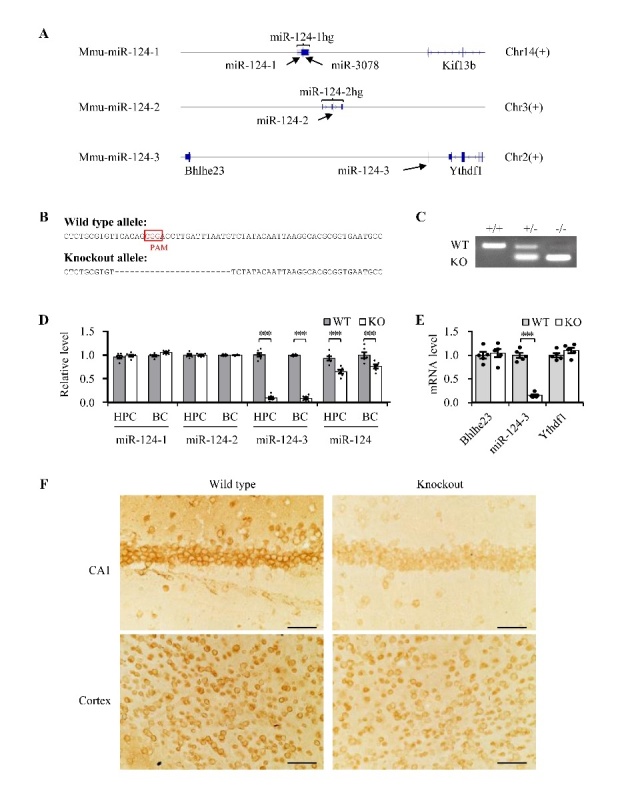
**MiR-124-3 knockout mice were established using the CRISPR-Cas9 system**. (**A**) Schematic diagram showed three pre-miR-124 loci (miR-124-1, miR-124-2, and miR-124-3).hg, host genes; Chr, chromosome. (**B**) DNA sequences of miR-124-3 genomic loci in both wild-type and knockout alleles. The red box indicates the protospacer adjacent motif (PAM) sequence. (**C**) Detection of wild-type (+/+) and homozygous (-/-) F1 mice using PCR products amplified from mouse tail genomic DNA. (**D**) qPCR analysis of pre-microRNA miR-124-1, miR-124-2, miR-124-3 and mature miR-124 in hippocampus (HPC) and cortex (BC) in both wild-type (+/+) and homozygous (-/-) mice. U6 was used as internal control and all data was normalized by wild-type group (mean ± SEM; n=6 mice per group; *P < 0.05, **P < 0.01, ***P<0.001; two-tailed Student’s *t*-test). (**E**) qPCR analysis of miR-124-3 upstream gene Bhlhe23 and downstream gene Ythdf1 mRNAs in wild-type (WT) and miR-124-3(-/-) (KO) mice. U6 was used as internal control and all data was normalized by wild-type group (mean ± SEM; n=6 mice per group; ***P<0.001; two-tailed Student’s t-test). (**F**) Representative images of miR-124 *in situ* hybridization staining in the hippocampal CA1 area and the parietal cortex of wild-type and miR-124-3(-/-) mice (Scale bar=40 μm).

MiR-124 is coded by three pre-miR-124 loci (miR-124-1, miR-124-2, and miR-124-3). Although the mature miRNA sequences are the same from the three miR-124 family members, the chromosomal location and precursor sequence differs (Fig. 2A and Supplementary Table 3). The genomic distribution analysis found that mouse miR-124-1 and miR-124-2 are both located in host genes (hgs), which are miR-124-1hg (ENSMUSG 00000097545) and miR-124-2hg (ENSMUSG0000010 0252), respectively. In contrast, miR-124-3 does not overlap with any other genes, which suggests that deletion of miR-124-3 has no effect on host genes (Fig. 2A). To examine the role of miR-124 deficiency in brain aging, we used the CRISPR-Cas9 system to generate miR-124-3 knockout mice that were deficient in miR-124 (Fig. 2B). Schematic diagrams of the sgRNA and the Cas9-encoding mRNA sequences are presented in Supplementary Figure 1. The sequence data showed that a 23-nucleotide sequence near the protospacer adjacent motif was deleted in the founder mice (Fig. 2B and 2C). Genotyping of the offspring confirmed the establishment of homozygous (miR-124-3(-/-)) and heterozygous (miR-124-3(-/+)) knockout mice (Fig. 2C). Neither the miR-124-3(-/-) nor miR-124-3(-/+) mice had evident signs of growth defects or infertility. Both the pre-microRNA and mature miRNA levels were analyzed to study the distribution of miR-124-3 in the brain. In miR-124-3(-/-) mice, the pre-microRNA of miR-124-3 was undetectable in the cortex and hippocampus of the brain (Fig. 2D). The mature miR-124 level was significantly reduced in the cortex and hippocampus (Fig. 2D). We also used *in situ* hybridization to evaluate the mature miR-124 levels. The results of this analysis supported the finding of a loss of mature miR-124 in miR-124-3(-/-) mice (Fig. 2F).

**Figure 3.**
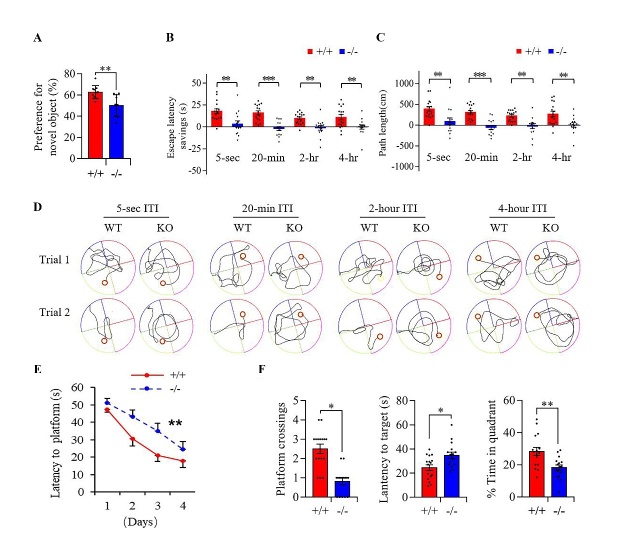
**Knockout of miR-124-3 resulted in learning and memory impairment in behavioral tests**. (**A**) Representative tracks of wild-type (+/+) and miR-124-3(-/-) mice in delayed matching-to-place water maze tasks of different inter-trial intervals (ITIs) (*upper panels*). Corresponding results for statistical analyses of time- (B) and length- (C) savings for different ITIs are presented in the lower panel. A larger difference means better performance. (mean ± SEM; n=16 mice per group; **P < 0.01, ***P<0.001; two-tailed Student’s t-test). (**D**) The time to find the hidden platform during the acquisition phase of Morris water maze standard hidden platform task (mean ± SEM; n=16 mice per group; **P < *0.01*; two-way ANOVA). (**E**) The numbers of crossings (*left panel*), the time required for the first crossing of the platform area (*medial panel*), and the time in the platform area (*right panel*) during probe trial phase of Morris water maze standard hidden platform task (mean ± SEM; n=16 mice per group; *P < 0.05, **P < *0.01*; two-tailed Student’s t-test). (**F**) The total exploration times (*left panel*) and preferences for a novel object (*right panel*) in the novel object recognition task (mean ± SEM; n=10 mice per group; *P*>0.05, **P < *0.01*; Student’s t-test, two-tailed).

To examine the effects of miR-124-3 disruption on the expression of other genes, we detected the mRNA level of its upstream and downstream genes in the chromosome. MiR-124-3 is in the intergenic region between two protein coding genes and is not in the intron or promoter regions of any gene (Fig. 2A). Therefore, the expression of genes nearby in the chromosome did not change after targeted deletion of miR-124-3 (Fig. 2E). In addition, we also compared the brain size and the neuron number of hippocampus and frontal cortex of adulthood miR-124-3(-/-) mice with wild-type mice. No obvious change of neurons number and brain size was detected in adult miR-124-3(-/-) mice (Supplementary Fig. 3). Taken together, these results indicated that miR-124 deficiency was successfully reproduced in miR-124-3(-/-) mice, without obvious effects on brain development.

### MiR-124 deficiency induced learning and memory impairment, but not motor coordination damage or depressive-like behavior

Impairment of learning and memory is a common feature of brain aging. Here we tested miR-124 deficiency-induced impairment of learning and memory using delayed matching-to-place (DMP) task and standard hidden platform task in a Morris water maze and novel object recognition task. In novel object recognition task, the discrimination index of miR-124-3(-/-) mice was significantly lower than that of wild -type mice, suggesting learning and memory impairment of miR-124-3(-/-) mice (Fig. 3A). The Morris water maze DMP task measures working memory. In this task, the mice were pre-trained for 5 days and then tested using four sessions with inter-trial intervals of 5 sec, 20 min, 2 h, or 4 h between trials 1 and 2 (Fig. 3D). Compared with the wild-type mice, the mean saving time and the mean saving path-length between trials 1 and 2 for the miR-124-3(-/-) mice were significantly less for all four sessions (Fig. 3B and 3C). This result suggested that the impairment of working memory in miR-124-3(-/-) mice. In the Morris water maze standard hidden platform task, the time to locate the hidden platform during the acquisition phase decreased gradually for miR-124-3(-/-) mice and wild-type mice (Fig. 3E). However, the decrease in time was slower for the miR-124-3(-/-) mice, compared with the wild-type mice (Fig. 3E). During the probe trial phase, the miR-124-3(-/-) mice spent significantly less time in the training quadrant, made fewer crossings through the platform area, and spent more time to first enter the platform area (Fig. 3F). The results revealed that the miR-124-3(-/-) mice had impairment of spatial reference learning and memory.

**Figure 4.**
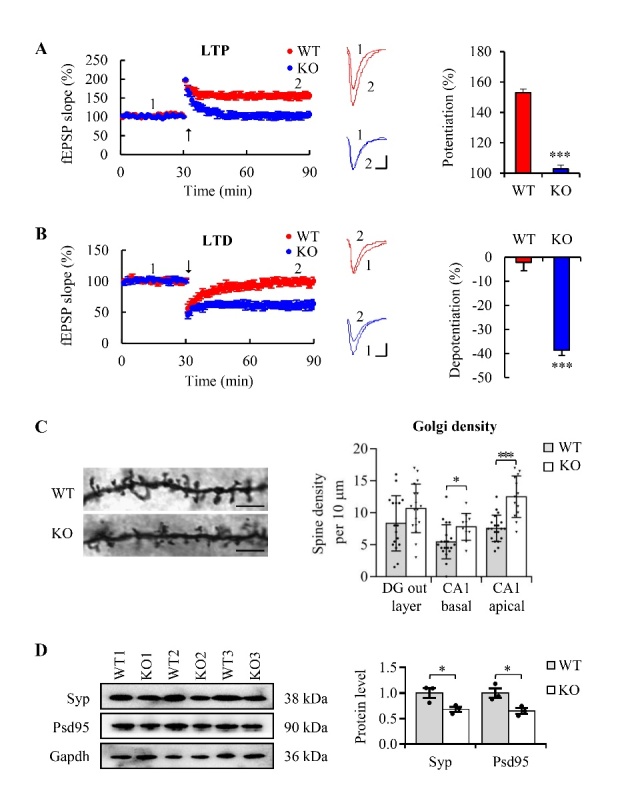
**MiR-124-3(-/-) mice showed synapse damage, LTP impairment and LTD enhancement in the hippocampus**. (**A**) Summery plots of mean normalized field EPSP slope (*left*), representative traces (*middle*) and quantitative analysis of long-term potentiation (LTP) (*right*) in hippocampus slices of miR-124-3(-/-)(KO) and wild-type(WT) mice. Arrow meant LTP induction (Scale bars=5ms, 0.2 mV; mean ± SEM; n=9 slices from 4 mice per genotypes; ***P<0.001; Mann-Whitney U test). (**B**) Summery plots of mean normalized field EPSP slope (*left*), example traces (*middle*) and quantitative analysis of long-term depression (LTD) (*right*) in hippocampus slices of miR-124-3(-/-)(KO) and wild-type(WT) mice. Arrow meant LTP induction (Scale bars=5 ms, 0.2 mV; mean ± SEM; n=10 slices from 4 mice per genotypes; ***P<0.001; Mann-Whitney U test). (**C**) Representative images of dendritic spine of neurons (*Right panel*) and spine densities (*left panel*) of the basal dendrites of dentate gyrus (DG) outer layer neurons and the basal and apical dendrites of CA1 neurons in both miR-124-3(-/-) and wild-type mice. (Scale bar=6 μm; mean ± SEM; n=5 mice per group; *P<0.05, ***P<0.001; Mann-Whitney U test). (**D**) Western blot and quantification of synaptophysin (Syp) and Psd-95 proteins in the hippocampus of miR-124-3(-/-) and wild-type mice. Gapdh was used as internal control and all data was normalized by wild-type group (mean ± SEM; n=3 mice per group; *P<*0.05*; the Mann-Whitney U test).

We also evaluated the motor coordination and balance of the miR-124-3(-/-) mice using footprint and rotarod tests; forced swimming test was used to evaluate depressive-like behavior. There were no statistically significant differences between the results for the miR-124-3(-/-) mice and the wild-type mice for stride length (Supplementary Fig. 2A), stride width (Supplementary Fig. 2A), latency on the rotarod (Supplementary Fig. 2B), immobility duration (Supplementary Fig. 2C), or swimming speed (Supplementary Fig. 2D).

Taken together, the results for the behavioral assessments indicated that miR-124 deficiency resulted in serious impairment of learning and memory, but did not reduce motor coordination or increase depression in the miR-124-3(-/-) mice.

**Figure 5.**
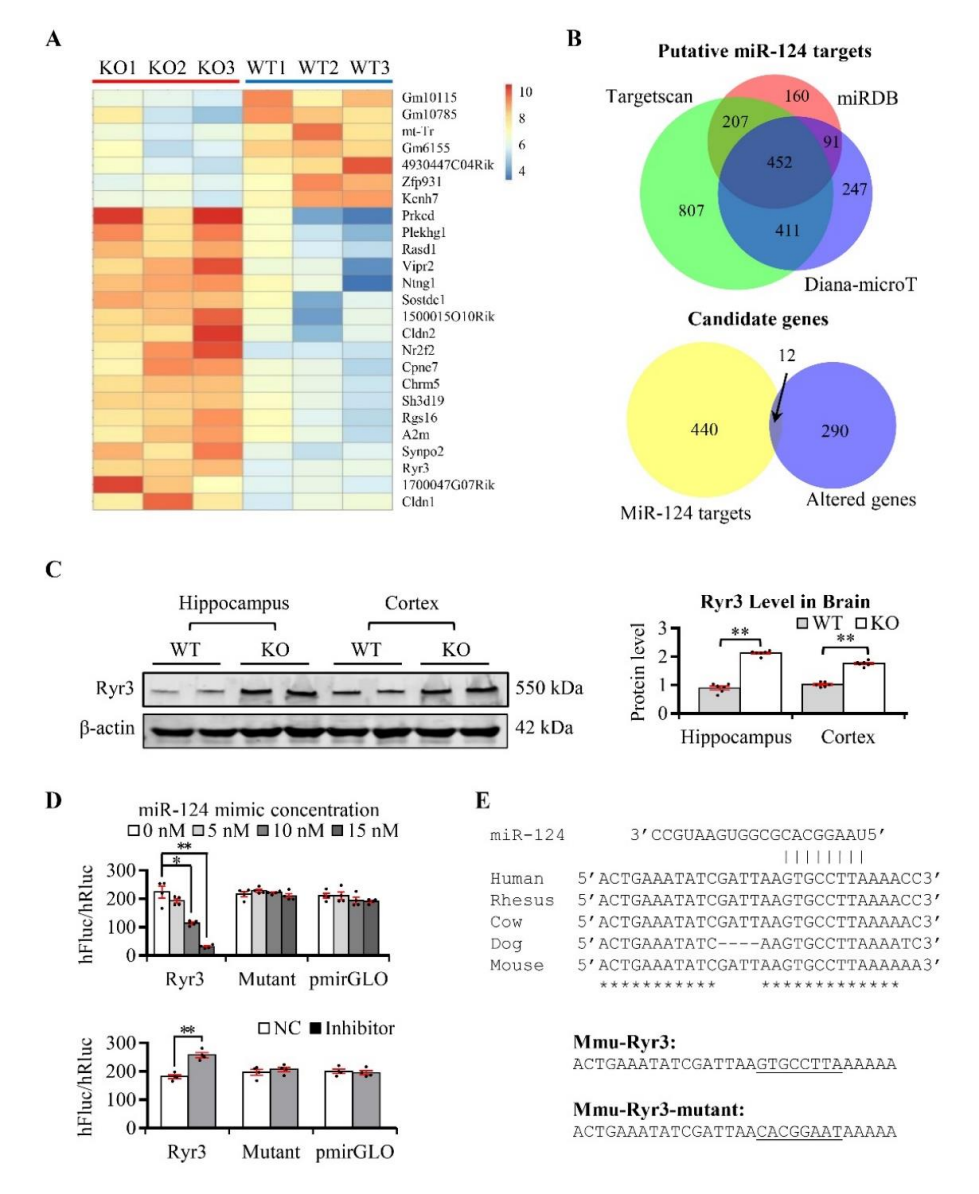
**Mir-124 targets RyR3 directly**. (**A**) Hierarchical clustering of systematic variations in the gene expression between miR-124-3(-/-) mice and wild type mice. (**B**) Venn diagram of the gene number of miR-124 putative targets from miRDB, TargetScan, and Diana-microT (*upper panel*), and the overlapped gene number between differentially expressed genes in miR-124-3(-/-) mice and miR-124 target genes (*lower panel*). (**C**) Western blot and quantification of Ryr3 in hippocampus and parietal cortex of miR-124-3(-/-) and wild-type mice. β-actin was used as internal control and all data was normalized by wild-type group (mean ± SEM; n=6 mice per group; *P < 0.05, **P < 0.01, ***P<0.001; two-tailed Student’s t-test). (**D**) MiR-124 targeted Ryr3 directly by luciferase assay: increasing amounts of miR-124-3 reduced the activity of the luciferase reporter with Ryr3-3'-UTR but had no effect on neither the mutant reporter nor the empty vector (*upper panel*); inhibition of miR-124-3 with miR-124-3 inhibitor could elevate the activity of the luciferase reporter with Ryr3-3'-UTR but no effect was observed in neither the mutant reporter nor the empty vector (*lower panel*) (mean ± SEM; n=4 per group; *P < 0.05, **P < 0.01; one-way *ANOVA* followed by Tukey *post hoc* test). (**E**) The sequences of miR-124 (*upper panel*) and its target sequences in the 3'-UTR of Ryr3 in different species. The seed sequences (*lower panel*) inserted into the 3’-UTR of luciferase reporter for both mmu-Ryr3 and mmu-Ryr3-mutant.

### MiR-124 down-regulation impaired synapse and LTP and enhanced LTD in the hippocampus

Neuroplasticity is characterized by changes in synapses, LTP (long-term potentiation) and LTD (long-term depression), which are the basis of learning and memory [34, 35]. To investigate the underlying mechanisms via which miR-124 deficiency induced the impairment of learning and memory, we examined the neural plasticity of the miR-124-3(-/-) mice. Electrophysiological results indicated that LTP was impaired and there was obvious LTD enhancement in sections of the hippocampi of the miR-124-3(-/-) mice (Fig. 4A and 4B). Correspondingly, Golgi staining showed that the basal and apical dendrite spine densities of the CA1 neurons were significantly decreased in the miR-124-3(-/-) mice (Fig. 4C, Supplementary Fig. 4). The expression of synapse marker synaptophysin and PSD-95 were also significantly reduced in the hippocampus of the miR-124-3(-/-) mice (Fig. 4D). These findings indicated that miR-124 down-regulation induced clinically detectable neuroplasticity alteration.

### Direct inhibition of miR-124 on RyR3 expression

To investigate the underlying molecular mechanisms via which miR-124 modulated learning and memory, a gene chip study was performed in three miR-124-3(-/-) mice and three of their littermates using the Affymetrix GeneChip which interrogated 28,853 genes with 770,317 distinct probes. Compared with wild type mice, the expression of 302 genes were significantly altered in the miR-124-3(-/-) mice (p<0.05, fold change>1.2, Supplementary Fig. 1). The heatmap of the top 25 differentially expressed genes was illustrated in Figure 5A, including 18 increased genes and 7 decreased genes (fold change>1,5). KEGG pathway (www.genome.jp/kegg/pathway.html) analysis indicated that these genes were predominantly involved in some neurogenic pathways, such as neuroactive ligand-receptor interaction, several synapses involved pathways, long-term potential and depression pathways and so forth (Supplementary Fig. 5A). To explore the direct targets of miR-124, we also compiled 452 putative target genes of miR-124, which were predicted using the three computational algorithms, miRDB, TargetScan, and Diana-microT (Supplementary Fig. 2); and then took the intersection between the putative miR-124 targeted genes and genes with altered expression in the miR-124-3(-/-) mice (Fig. 5B). There were 12 differentially expressed genes were putative miR-124 target genes (Fig. 5b). Finally, we selected nine genes that have important roles in brain aging and SAD and used western blot to examine their expression levels in the miR-124-3(-/-) and wild-type mice (Supplementary Fig. 5). Four proteins, RyR3, Ptbp1, Map3k3 and P65 increased significantly in the miR-124-3(-/-) mice (Supplementary Fig. 5B and C). Ptbp1, Map3k3 and P65 were reported to be targeted by miR-124 before. However, there is no information about the relationship between miR-124 and RyR3 (Ryanodine receptor 3).

To examine the effect of miR-124 on RyR3 regulation, we produced a luciferase reporter with the predicted target region from the 3'-UTR of RyR3; and we also mutated the seed sequence to generate a negative control (Fig. 5F). The luciferase reporter and different concentrations of miR-124 mimics were co-transfected into HEK293 cells. The results indicated that in the reporter derived from the RyR3-targeted region, miR-124 suppressed luciferase activity in a dose-dependent manner (Fig. 5E). As negative controls, luciferase activity of the cells containing the mutant 3'-UTR or the empty pmirGLO vector was not affected by miR-124 (Fig. 5D *upper*). In contrast, knockdown of miR-124 would increase RyR3 expression since the luciferase activity of the reporter derived from the RyR3-targeted region significantly increased after treatment of miR-124 inhibitor, but no changes were observed in the mutant 3'-UTR or the empty pmirGLO vector (Fig. 5D *lower*). Alignment of RyR3 3'-UTR sequences in different species also revealed that the miR-124 binding site was conserved in vertebrates (Fig. 5E *upper*). To confirm protein level of RyR3 on the endoplasmic reticulum was up-regulated, we collected membrane of hippocampus by ultracentrifugation and then found RyR3 level also increased (Supplementary Fig. 6B). Taken together, these results indicated that miR-124 targeted RyR3 directly and that the miR-124 deficiency in the miR-124-3(-/-) mice resulted in RyR3 increases in the hippocampus.

### Knockdown of RyR3 partly reversed the impairment of cognitive function, LTP, and LTD and the dysregulation of Ca^2+^ homeostasis in miR-124-3 knockout mice

**Figure 6.**
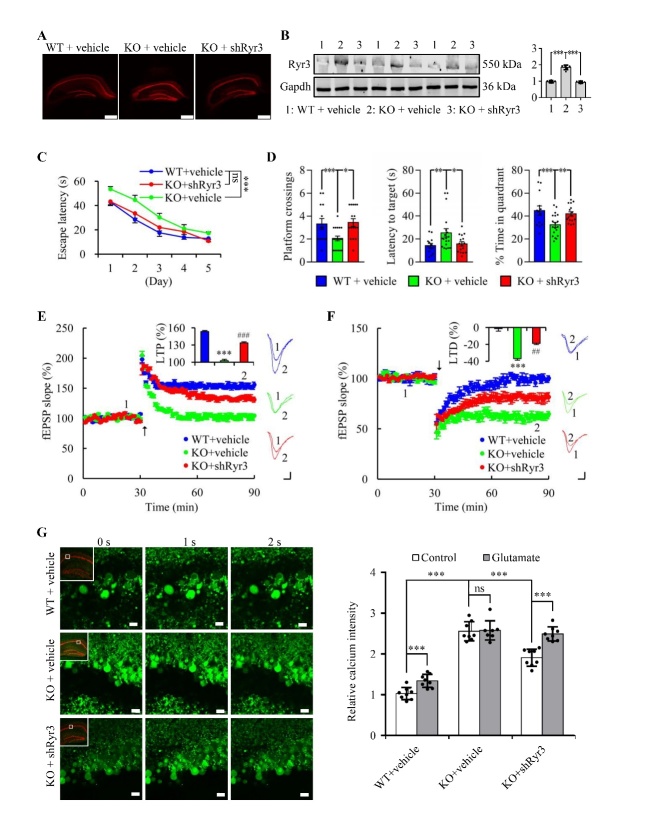
**Suppression of RyR3 expression partially reversed the impairment of cognitive function, LTP, and LTD and the dysregulation of Ca^2+^ homeostasis in miR-124-3 knockout mice**. (**A**) Representative fluorescence images of hippocampus sections 28 days after the injection of AAV-mCherry-ShRyr3 or vehicle (Scale bars=0.5 mm). (**B**) Representative western blot and statistical analysis of RyR3 proteins in hippocampus of wild-type (WT) and miR-124-3(-/-) (KO) mice after injection of AAV-mCherry-ShRyr3 or vehicle. Gapdh was used as internal control and all data was normalized by wild-type group. (mean ± SEM; n=3 mice per group; *P < 0.05, **P < 0.01, ***P<0.001; Mann-Whitney U test). (**C**) The time to find the hidden platform during the acquisition phase of Morris water maze standard hidden platform task (mean ± SEM; n=13, 19 and 16 mice for WT+vehicle, KO+vehicle and KO+ShRyr3 group, respectively; **P < 0.01, ***P<0.001, ns =no significance; two-way ANOVA followed by Tukey *post hoc* test). (**D**) The numbers of crossings (*left panel*), the time required for the first crossing of the platform area(*medial panel*), and the time in the platform area (*right panel*) during probe trial phase of Morris water maze standard hidden platform task (mean ± SEM; n=13, 19 and 16 mice for WT+vehicle, KO+vehicle and KO+ShRyr3 group, respectively; *P < 0.05, **P < 0.01, ***P<0.001; one-way ANOVA followed by Tukey *post hoc* test). (**E**) Summery plots of mean normalized field EPSP slope, example traces and quantitative analysis of long-term potentiation (LTP) in hippocampus slices of miR-124-3(-/-) (KO) and wild-type (WT) mice after injection of AAV-mCherry-ShRyr3 or vehicle. Arrow meant LTP induction (Scale bars=5 ms, 0.2 mV; mean ± SEM; n=14 slices from 6 mice per genotypes; ***P<0.001; one-way ANOVA followed by Tukey *post hoc* test.). (**F**) Summery plots of mean normalized field EPSP slope, example traces and quantitative analysis of long-term depression (LTD) in hippocampus slices of miR-124-3(-/-)(KO) and wild-type(WT) mice after injection of AAV-mCherry-ShRyr3 or vehicle. Arrow meant LTP induction (Scale bars=5 ms, 0.2 mV; mean ± SEM; n=13 slices from 6 mice per genotypes; ***P<0.001; one-way ANOVA followed by Tukey *post hoc* test). (**G**) Representative images (*left panel*) and quantification (*right panel*) of calcium fluorescence by Fluo-4, AM in hippocampus slice of WT+vehicle, KO+vehicle and KO+shRyr3 mice before (0 s), and after (1s and 2s) glutamate (10 μM) stimulus(Scale bars=20μm, mean ± SEM; n= 8 slices from 4 mice per group; **P < 0.01, ***P<0.001, ns =no significance; two-way ANOVA followed by Tukey *post hoc* test).

To support the hypothesis that RyR3 upregulation induced by hippocampal miR-124 deficiency was the key factor affecting learning and memory decline in miR-124-3 (-/-) mice, we knocked down hippocampal RyR3 of miR-124-3(-/-) mice by injecting AAV9 with short hairpin RNAs (Fig. 6A) and then examined the changes in learning and memory. We found that the RyR3 protein level in the hippocampus of AAV9-shRNA injected miR-124-3(-/-) mice was significantly lower, compared with miR-124-3(-/-) mice injected control virus (Fig. 6B). The Morris water maze test results indicated that reduction in RyR3 expression in the hippocampus could significantly rescue the impairments in spatial learning and memory (Fig. 6C and D). Electrophysiological detection and calcium fluorescence of a hippocampus section also revealed that in the AAV9-shRNA injected miR-124-3 (-/-) mice, the LTP impairment and Ca^2+^ homeostasis dysregulation was partially rescued, and the LTD induction was depressed (Fig. 6F-G).

### MiR-124 deficiency induced neuroinflammation

Increased neuroinflammation is an important feature of aged brains. This inflammation is commonly characterized by the levels of microglial activation and inflammatory factors [6, 36]. Iba-1 immunostaining revealed that there was no significant difference in mean hippocampal microglial cell number between the miR-124-3(-/-) mice and the wild-type mice (Supplementary Fig. 7). However, in the miR-124-3-/- mice, the percentage of activated microglia was significantly higher than in the wild-type mice (Supplementary Fig. 7). Consistent with the changes in microglial activation, the mRNA levels of IL-1β and TNF-α were also significantly higher in the miR-124-3(-/-) mice than in the wild-type mice (Supplementary Fig. 7). These results indicated that miR-124 deficiency induced obvious neuroinflammation. We also observed that apoptosis-related factors, Bax, pro-caspase 1 and gsdmd, were up-regulated in miR-124-3(-/-) mice (Supplementary Fig. 6). In addition, aging marker, p16lnk4a, also increased in miR-124-3(-/-) mice (Supplementary Fig. 6).

### MiR-124 deficiency facilitated β-amyloid-induced neuron apoptosis

Neurotoxicity of β-amyloid has been proposed as a cause of sporadic Alzheimer’s disease (SAD) [37, 38]. To examine the role of miR-124 deficiency of aged brain in SAD pathogenesis, we injected exogenous Aβ1-42 into the hippocampi of miR-124-3(-/-) mice and wild-type mice (Supplementary Fig. 8A). We then analyzed the differences in neuron apoptosis. Compared with the wild-type mice, Aβ1-42 induced more apoptotic cells (Supplementary Fig. 8A and B) and a wider range of apoptosis (Supplementary Fig. 8A and C) in the hippocampi of miR-124-3(-/-) mice. This result suggested there was higher neuronal vulnerability to Aβ1-42 in miR-124-3(-/-) mice.

## DISCUSSION

In this study, we found age-dependent loss of miR-124 in human and rat brains. Because miR-124 is highly enriched in neurons, aging-associated neuron number reducing could be responsible for the loss of miR-124. However, the results of *in situ* hybridization supported the miR-124 loss in neurons, which indicated that the age-dependent loss of brain miR-124 was not due to a secondary effect of neuron loss. MiR-124 is coded by three genes, miR-124-1, miR-124-2, and miR-124-3. The genomic distribution analysis found that miR-124-3 did not overlap with any other host genes (hgs); it was obviously different from miR-124-1 and miR-124-2. Both miR-124-1 and miR-124-2 are located in host genes (miR-124-1hg: ENSMUSG00000097545; miR-124-2hg: ENSMUSG00000100252), and knockout of miR-124-1 induces obvious developmental brain deficits [39]. Thus, we chose to construct miR-124-3(-/-) mice to mimic clinical miR-124 deficiency in aged brain and detected the effects of miR-124 deficiency on brain. As we expected, the miR-124-3(-/-) mice had no obvious deficits in brain size or neuron number until adulthood (Supplementary Fig. 3), but showed significant miR-124 deficiency (Fig. 2). In addition, the miR-124 deficiency in our miR-124-3(-/-) mice reproduced the core phenotypes of brain aging (i.e., cognitive deficits, synapse loss, abnormal electrophysiology, and neuroinflammation (Fig. 3 and 4). And we also found that programed cell death related proteins (Supplementary Fig. 6A) and aging marker, p16lnk4a (Supplementary Fig. 6C and D), increased in the hippocampus of miR-124-3(-/-) mice, and exogenous supply of miR-124 could reverse the hippocampal impairment of LTP in aged mice (Supplementary Fig. 8D-F). Combined with the fact that miR-124 expression is easily regulated by environmental factors, our results suggested that age-dependent loss of miR-124 plays an important role in ARCD as a downstream cell-signaling node, and miR-124 could be a tractable epigenetic molecule for anti-ARCD drug development.

Ryanodine receptors (RyRs) are intracellular calcium-release channels located on the endoplasmic reticulum of all cells [40]. RyRs are a major component of control of the intracellular Ca^2+^ levels. Three distinct RyR protein isoforms (RyR1, RyR2 and RyR3) are expressed in a spatial manner, and RyR3 is especially expressed in the brain [41]. During brain aging, RyR3 expression gradually increases [40] and leads to intracellular Ca^2+^ dyshomeostasis [42, 43]. The latter causes the disbalance of downstream calcium-dependent phosphorylases or phosphatases (e.g.,Ca^2+^/calmodulin-dependent protein kinase and calcineurin) [44, 45]. The consequences of these changes include impairment of learning and memory [46], synapse loss [47], neuroinflammation [48], neuron loss [49], impaired LTP [34], and enhanced LTD [50]. Ca^2+^ overload in cytoplasm can significantly increase the neurotoxicity of amyloid-β oligomers in aged cultured hippocampal neurons [51]. Our study found that a deficiency of brain miR-124 resulted in upregulation of the RyR3 gene via direct interaction. The cognitive deficits and intracellular Ca^2+^dyshomeostasis in the miR-124-3 knockout mice were ameliorated by knockdown of RyR3 expression using RNAi (Fig. 6). Thus, we proposed a new important downstream mechanism of ARCD,which was miR-124 deficiency-induced Ca^2+^ dyshomeostasis via RyR3. This finding differs from those of previous studies that miR-124 has a role in AD by directly targeting PTPN1, calpain 1, and BACE1 and blocking the progressive buildup of β-amyloid [52-54].

Age is the most accepted risk factor of SAD [55-57]. However, the molecular underpinnings shared by brain aging and SAD remain elusive. Our study detected age-dependent loss of brain miR-124 which induced core phenotypes of brain aging. Previous studies have reported miR-124 loss in patients with Alzheimer’s disease [14, 52, 53, 58] and in a fly model of Alzheimer’s disease [59]. Viral-mediated miR-124 overexpression can inhibit β-amyloid production [52, 60] and relieve the cognitive defects of APP/PS1 transgenic mice [60]. In addition, we found that MiR-124 deficiency enhanced the vulnerability of neurons to β-amyloid neurotoxicity, probably facilitating the on-set of Alzheimer’s disease (Supplementary Fig. 8). These results indicate miR-124 deficiency is an important pathomechanism shared by ARCD and SAD. MiR-124 is a possible target for the prevention and treatment of SAD at the preclinical or prodromal stages.

In our study, brain miR-124 levels progressively decreased during brain aging. The underlying mechanism remains unclear. Chronic stress [19], exercise [20], and chronic cerebral hypoxia [21] all significantly decrease hippocampal miR-124 expression. MiR-124 expression in activated microglial cells also decreases in mice with experimental autoimmune encephalomyelitis [22]. Viral proteins, such as HIV-1 Tat, can also decrease the expression of microglial cell miR-124 via modulation of DNA methylation [61]. Taken together, these findings suggested that miR-124 down-regulation during cognitive aging is possibly due to the effects of certain environmental factors. However, the exact molecular mechanisms via which environmental factors modulate miR-124 expression need further investigation.

In conclusion, we found that miR-124 was downregulated in the aged subjects. Our findings revealed a novel mechanism via which miR-124 deficiency depresses RyR3 expression to induce cognitive deficits in aged brain.

## Supplementary Materials

The Supplementary data can be found online at: www.aginganddisease.org/EN/10.14336/AD.2022.0204.


