# Supplementary material for: Age-related Loss of miR-124 Causes Cognitive Deficits via Derepressing RyR3 Expression
Source: Aging Dis. 2022 Oct 1;13(5):1455–70. doi: 10.14336/AD.2022.0204 (PMC9466975; doi:10.14336/AD.2022.0204)
Supplement: Supplementary file 1 [file AD-13-5-1455-s.pdf]

SUPPLEMENTARY DATA

Age-related Loss of miR-124 Causes Cognitive Deficits *via* Derepressing RyR3 Expression

Kai Liu[1,3,6], Yongjia Yin[2], Yuan Le[1], Wen Ouyang[1], Aihua Pan[4], Jufang Huang[4], Zhongcong Xie[5], Qubo Zhu[2*], Jianbin Tong[1,3*]

© 2022. Lui K et al. Published online at http://www.aginganddisease.org/EN/10.14336/AD.2022.0204

SUPPLEMENTARY DATA

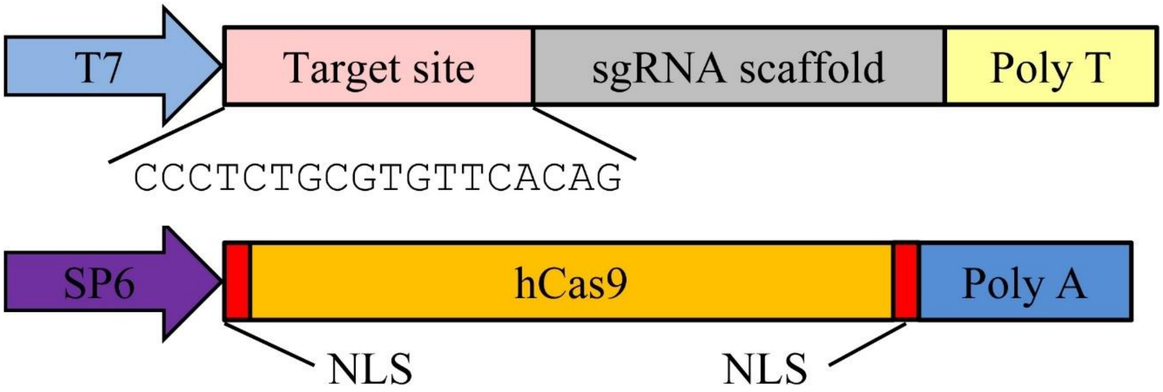

Supplementary Figure 1. RNA constructs of the Cas9/RNA system used in this study. T7, T7 promoter; SP6, SP6promoter; NLS, nuclear localization signal.

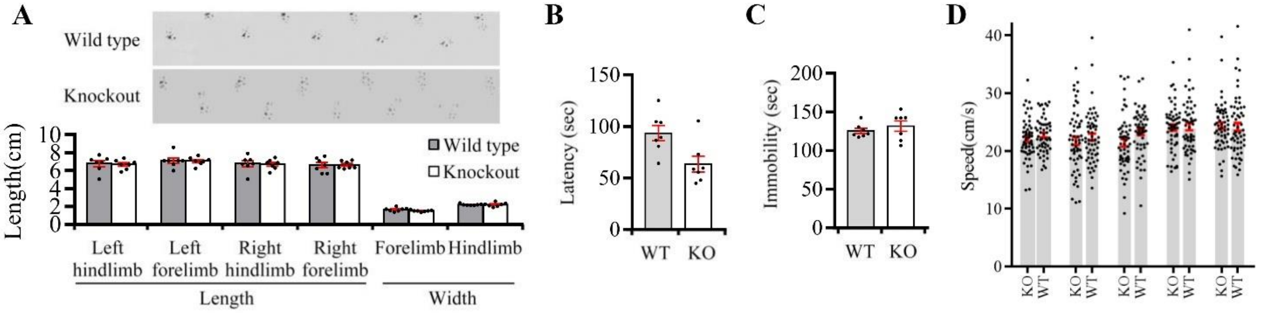

Supplementary Figure 2. Motor activity of wild-type (+/+) and homozygous (-/-) mice. (A) Footprints were recorded for the two types of mice. Stride lengths were measured for the left hindlimb, left forelimb, right hindlimb, and right forelimb; the forelimb and hindlimb stride widths were the same between the two genotypes. (N=7 mice per group. Two-sided Student's t-tests (left hindlimb: t=0.102, *P*=0.9202; left forelimb: t = 0.1405, *P*=0.8903; right hindlimb: t=0.4742, *P*=0.6427; right forelimb: t=0.8306, *P*=0.4201; forelimb: t=0.7966, *P*=0.4390; hindlimb: t=1.685, *P*=0.1142) or Mann-Whitney U tests.). **(B)** In the rotarod tests, the latencies to fall from the rotated rods were similar for all genotypes (N=7 mice per group. latency: t=2.564, P=0.0854; Two-sided Student's t-tests). **(C)** In the forced swimming tests, the immobility durations of the two genotypes of mice were recorded to assess the depression-like moods of these mice. (N=7 animals per group, t=1.04, *P*=0.112, two-sided Student's t-tests) **(D)** Swimming speed of the two genotypes of mice for 5 days. (* *P* <0.05, ** *P* <0.01, *** *P* <0.001)

SUPPLEMENTARY DATA

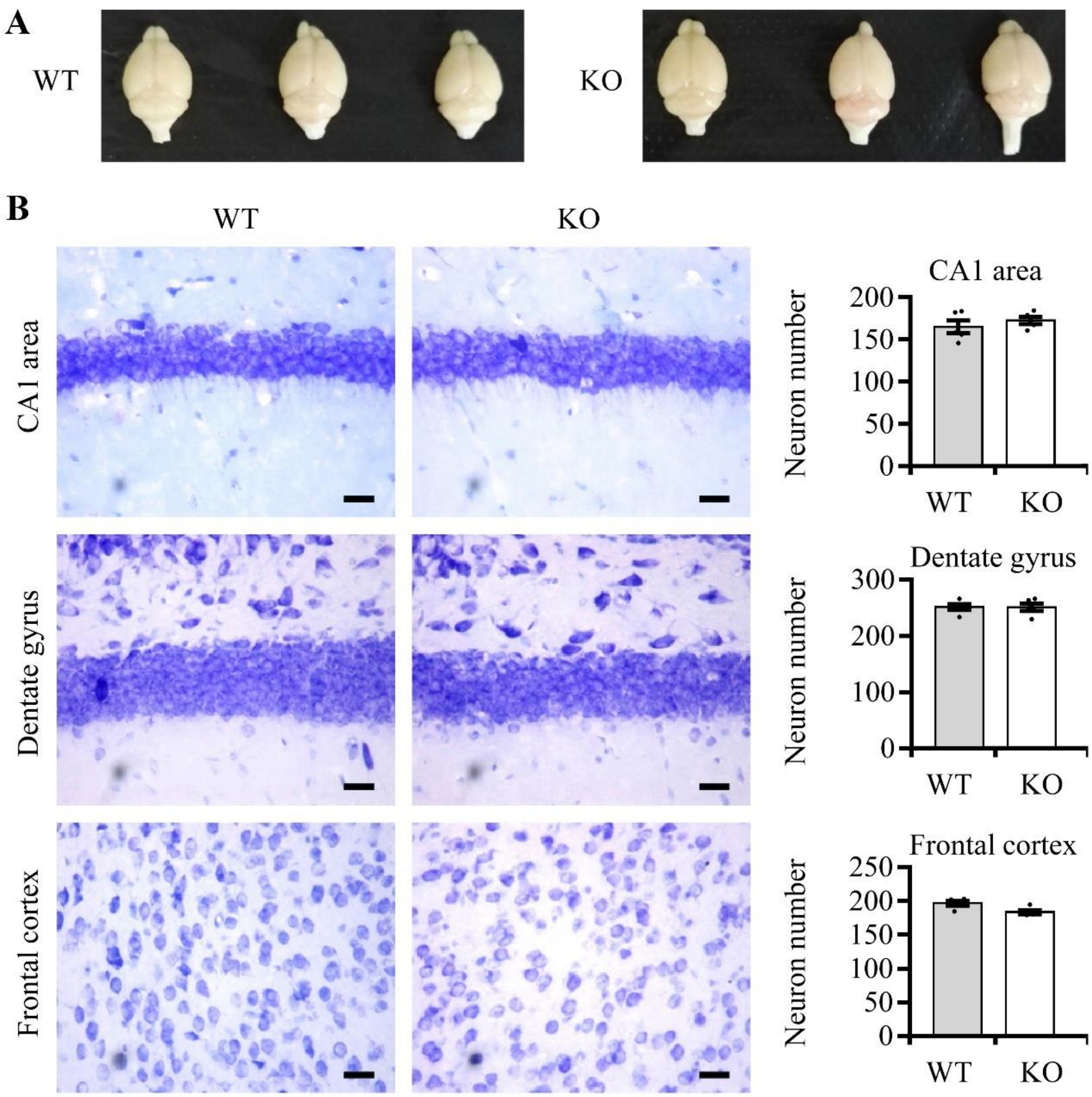

Supplementary Figure 3. Brain size and neuron number in the hippocampus and frontal cortex of wild-type and miR-124-3 (-/-) mice (3–4 months). (**A**) *Ex vivo* images of the brains revealed that the brain sizes of both the wild-type and miR-124-3(-/-) mice were similar. (**B**) Representative images of hippocampal sections stained with Nissl indicated that the neuron densities of the miR-124-3(-/-) mice and wild-type mice were the same. Scale bar, 40 μm. Right panel shows the quantification analysis of the neuronal counts in the hippocampal CA1, dentate gyrus and frontal cortex regions (n=4 for each group. Results are mean ± SEM values. The data were analyzed using Mann-Whitney U tests (CA1 area: t=1.052, *P*=0.2982; DG: t = 0.08838, *P*=0.9307; FC: t=2.08, *P*=0.0711)).

© 2022. Lui K et al. Published online at http://www.aginganddisease.org/EN/10.14336/AD.2022.0204

SUPPLEMENTARY DATA

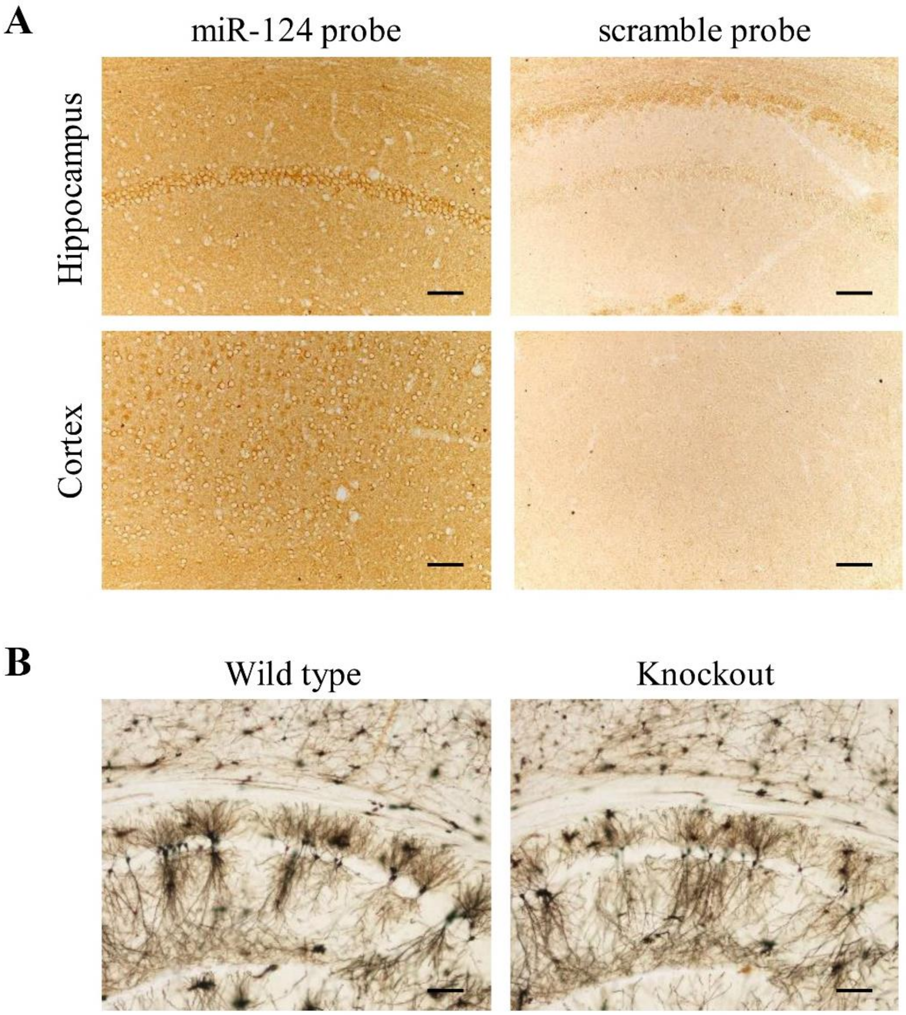

Supplementary Figure 4. *In situ hybridization* by scramble probe and miR-124 probe (Scale bar, 25 µm) **and Golgi staining of the CA1 of wild type and miR-124-3(-/-) mice** (Scale bar, 80 µm)**.**

© 2022. Lui K et al. Published online at http://www.aginganddisease.org/EN/10.14336/AD.2022.0204

SUPPLEMENTARY DATA

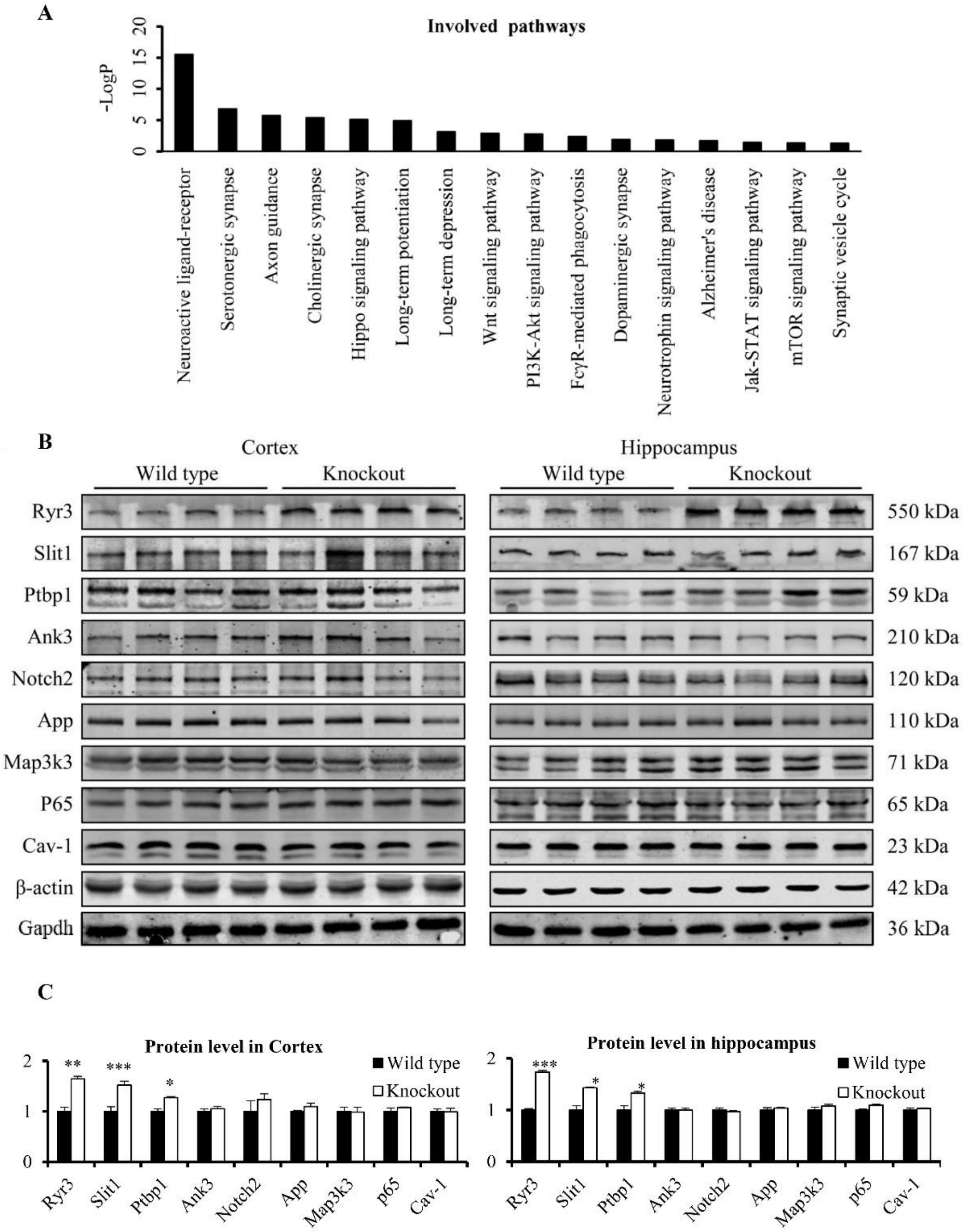

Supplementary Figure 5. (A) KEGG pathway analysis of significantly altered gene in the miR-124-3(-/-) mice compared to wild type mice. **(B)** Western blot and quantification of downstream gene proteins in hippocampus and parietal cortex of miR-124-3(-/-) and wild-type mice. β-actin and GAPDH was used as internal control (mean ± SEM; n=4 mice per group; *P ＜ 0.05, **P ＜ 0.01, ***P＜0.001; Mann-Whitney U tests).

© 2022. Lui K et al. Published online at http://www.aginganddisease.org/EN/10.14336/AD.2022.0204

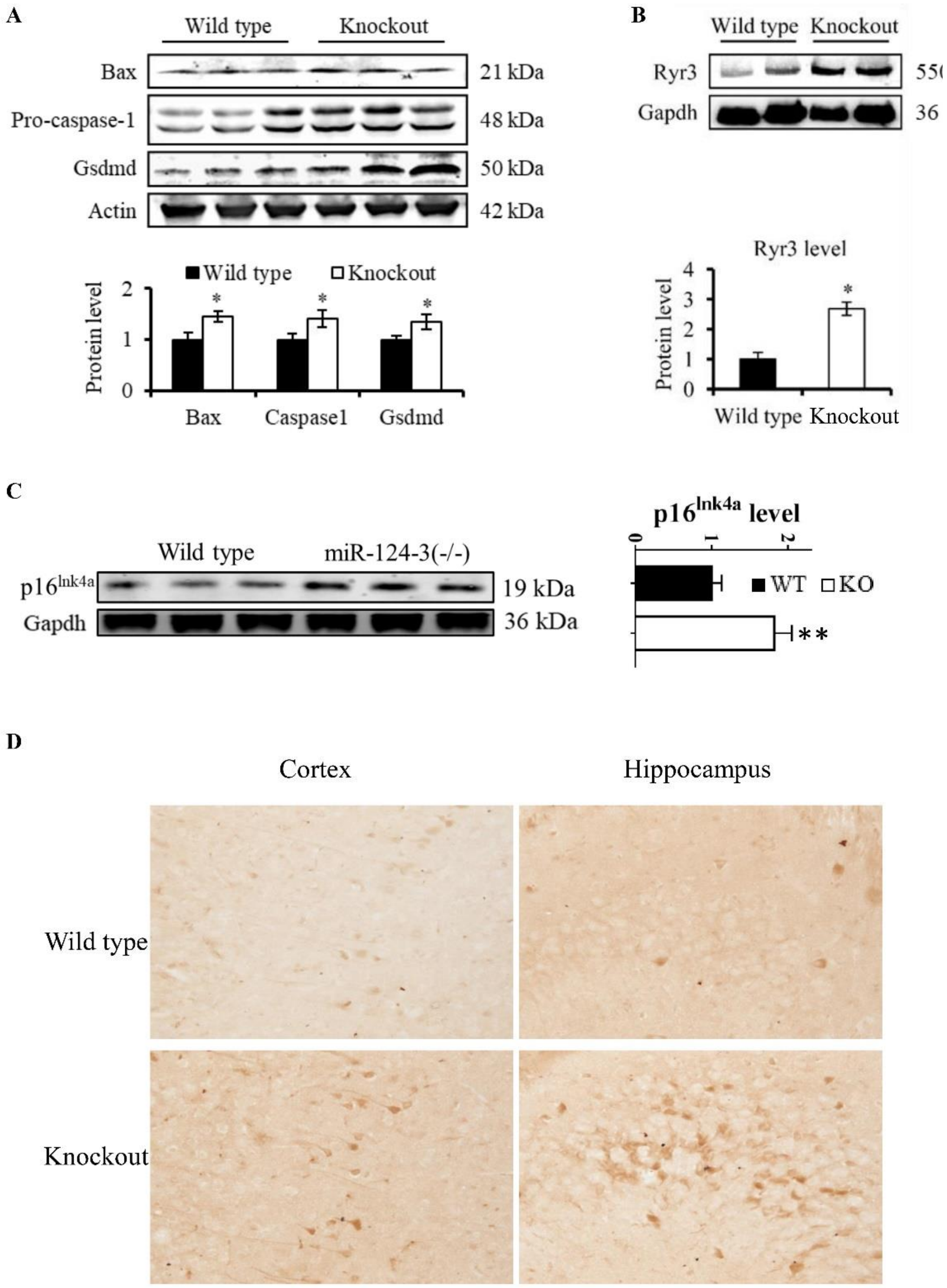

Supplementary Figure 6. Apoptosis and pyroptosis factors increased in miR-124-3(-/-) mice. (**A**)Protein levels of Bax, caspase-1, and gasdermin D(GSDMD) were detected using western blot; β-actin was used as an internal control. The statistical plot of the western blot bands, upper panel. (**B**) Protein levels of RyR3 (isolated using ultracentrifugation) on membranes and statistical plots of the western blot bands. (**C**) Protein levels of p16^{lnk4a} and statistical plots of the western blot bands. (**D**) Representative images of immunostaining for p16^{lnk4a}. (The results are mean ± SEM values, data analyzed using Mann-Whitney U tests). (* *P* <0.05, ** *P* <0.01, *** *P* <0.001).

© 2022. Lui K et al. Published online at http://www.aginganddisease.org/EN/10.14336/AD.2022.0204

SUPPLEMENTARY DATA

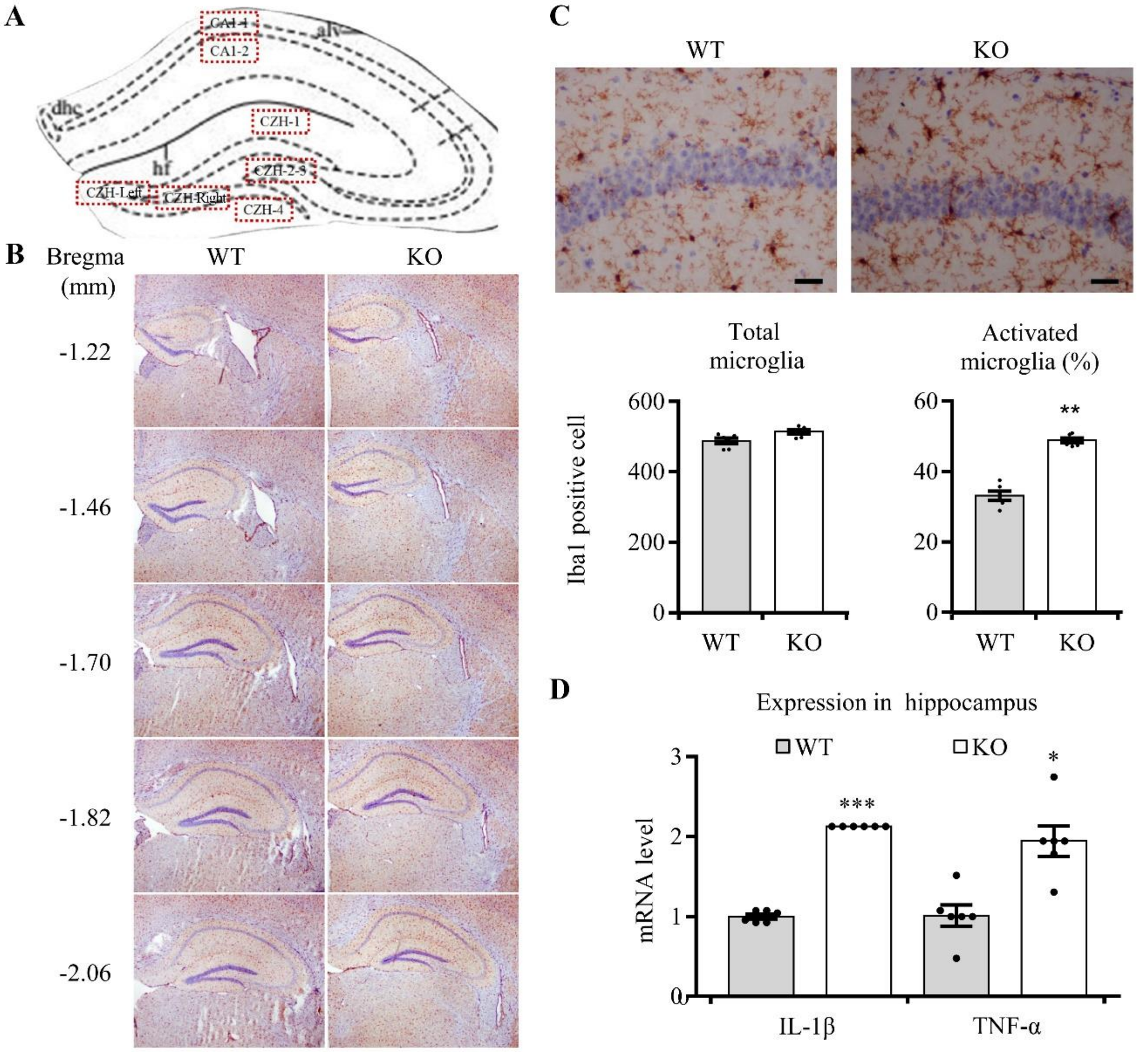

Supplementary Figure 7. Neuroinflammation was enhanced in the brains of miR-124-3(-/-) mice. (A) Schematic diagram of seven selected areas in the hippocampal CA1 and dentate gyrus regions. **(B)** Images of regions of the hippocampus were taken at the levels of -1.22, -1.46, -1.70, -1.82, and -2.06 mm, relative to the bregma; all were taken at the same magnification (40× objective lens). **(C)** Upper panel, representative images showing immunostaining for Iba1 in the hippocampus. A quantitative analysis (lower panel) found that activated microglia was significantly increased in the miR-124-3(-/-) mice. **(D)** qPCR analysis of proinflammatory cytokine mRNA levels for TNF-α and IL-1β in the hippocampus between two groups; β-actin was used as an internal control. (The results were presented as mean ± standard deviation values. N=6. Total microglia: t=1.662, P =0.1719; active microglia: t=7.186, P =0.002; mRNA level: IL-1β:t=26.85, P <0.0001; TNF-α: t=2.876, P =0.0452). (* *P <0.05*, ** *P <0.01*, *** *P <0.001*)

© 2022. Lui K et al. Published online at http://www.aginganddisease.org/EN/10.14336/AD.2022.0204

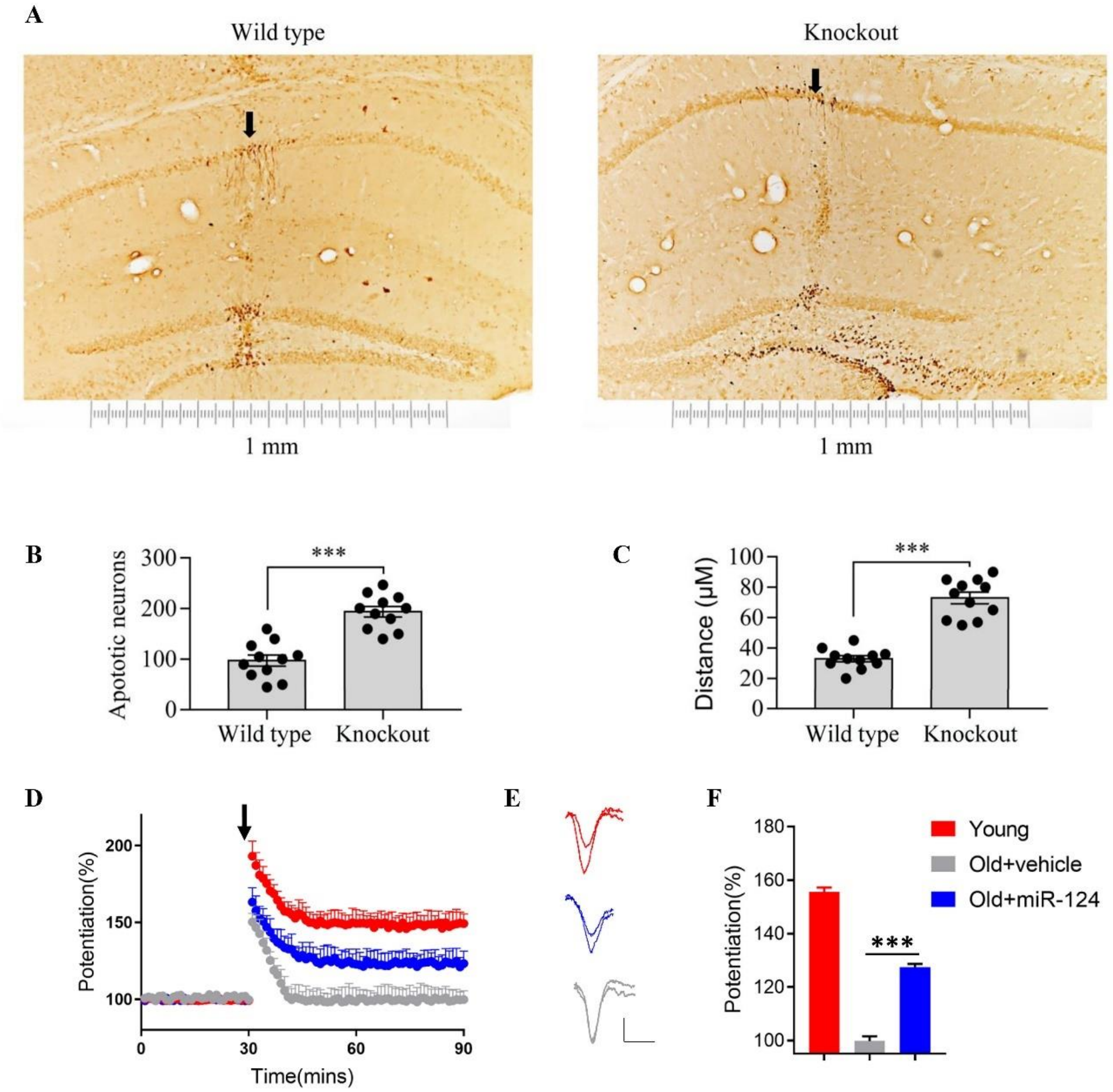

Supplementary Figure 8. Aβ1-42 induced more serious neuron apoptosis in the hippocampus of miR-124-3(-/-) mice. (**A**) Representative images of hippocampal neuron apoptosis of wild-type (WT) and miR-124-3(-/-) (KO) mice 3 days after exogenous Aβ1-42 injection. Arrow meant needle track. (**B**) Quantification of apoptotic cells in the hippocampus of wild-type (WT) and miR-124-3(-/-)(KO) mice 3 days after exogenous Aβ1-42 injection(mean ± SEM; n=11 sections from 4 mice per genotypes; ****P*＜*0.001*; Mann-Whitney U tests). (**C**) The farthest distance from needle track to apoptotic cells disappearance in the hippocampus of wild-type (WT) and miR-124-3(-/-)(KO) mice3 days after exogenous Aβ1-42 injection (mean ± SEM; n=11 sections from 4 mice per genotypes; ****P* ＜*0.001*; Mann-Whitney U tests). (**D**) Summery plots of mean normalized field EPSP slope (Arrow meant LTP induction), example traces **(E)** and quantitative analysis of long-term potentiation (LTP) **(F)** in hippocampus slices of Young (3 month), Old (20 month) +Vehicle and Old (20 month) +MiR-124 group. (Scale bars=10 ms, 1 mV; mean ± SEM; n=10 slices from 3 mice per group; ***P＜0.001; Mann-Whitney U tests).

SUPPLEMENTARY DATA

Supplementary Table 1. Sequences of PCR primer sets used in this study.

Primer name	Access number	Primer sequence (direction 5′–3′)
miR-124-3<br>Wild-type	NC_000068.7	Forward: CTCTGCGTGTTCACAGCG<br>Reverse:CCTTCTCGTGACGTCCTAGG
miR-124-3<br>Knock-out	NC_000068.7	Forward: GCCCCTCTGCGTGTTCTATA<br>Reverse: ATTGTTCGCCGGATTTGTCC
miR-124		RT-Primer：<br>GTCGTATCCAGTGCAGGGTCCGAGGTATTCGCACTGGATAC GACGGCATT<br><br>Forward: ATAATTCGGTAAGGCACGCGGTG<br>Reverse: ATCCAGTGCAGGGTCCGAGG
Mmu-pre-miR-124-3	NC_000068.7	Forward: TGCGTGTTCACAGCGGAC<br>Reverse: CGCGTGCCTTAATTGTATAGACA
Mmu-pre-miR-124-1		Forward: AGGCCTCTCTCTCCGTGTTC<br>Reverse: CCCATTCTTGGCATTCACC
Mmu-pre-miR-124-2		Forward: AGACTCTGCTCTCCGTGTTC<br>Reverse: CGTAGGCTCCGCTCTTGG
Mmu-U6	NM_001191004.1	Forward: GGCAGGAACATGGCAGCATC<br>Reverse: GCGTGGGCTTTAGCTTGTCC
Mmu-IL-1β	NM_008361.4	Forward: GCCCATCCTCTGTGACTCAT<br>Reverse: AGGCCACAGGTATTTTGTCG
Mmu-TNF-α	NM_013693.3	Forward: ATGCACCACCATCAAGGACTCAA<br>Reverse: ACCACTCTCCCTTTGCAGAACTC
Rat-U6	NM_001126085.1	Forward: CTCAGGTTTCCTGCCTCCCA<br>Reverse: CCTCGGTAATCCACGCCAGA
Hsa-U6	NM_007080.2	Forward: AAAGCAAATCATCGGACGACC<br>Reverse: GTACAACACATTGTTTCCTCGGA

Supplementary Table 2. Detailed information about the statistical analyses associated with each Figure.

**Fig. #**	**Compare (group size; n)**	**Passed normality test (Yes/No)**	**Statistical method**	***P*-value**	**T-value**	**F-value**
**Fig. 1C**	n =6,8 and 9 for >60, 50-60 and <50 group	Yes	one-way ANOVA followed by Tukey *post hoc* Test	*P*<0.0001		$F_{(2, 21)} = 29.15$
**Fig. 1E**	*in situ* hybridization of miR-124 in the cortex and hippocampus of different ages of rats.	Yes	one-way ANOVA followed by Tukey *post hoc* test.	*P<0.0001*		Cortex: $F_{(3, 28)} = 279.7$
	2months vs 12months			*P <0.001*		
	12months vs 24months			*P*=0.0235		
	24months vs 33months			*P*=0.0023		
				P<0.0001		Hippocampus CA1: $F_{(3, 32)} = 594.4$
	2months vs 12months			*P <0.001*		
	12months vs 24months			*P=0.0003*		
	24months vs 33months			*P=0.0053*		
**Fig. 1F**	miR-124 in the cortex and hippocampus of rats (n=6 for each age-group)	Yes	one-way ANOVA followed by Tukey *post hoc* Test	*P<0.0001*		Cortex: $F_{(3, 20)} = 34.83$
	2months vs 12months			*P=0.0012*		
	12months vs 24months			*P=0.0401*		
	24months vs 33months			*P*=0.0020		
				P<0.0001		Hippocampus: $F_{(3, 28)} = 211.9$
	2months vs 12months			*P*=0.0016		

© 2022. Lui K et al. Published online at http://www.aginganddisease.org/EN/10.14336/AD.2022.0204

SUPPLEMENTARY DATA

	12months vs 24months			*P*=0.0027		
	24months vs 33months			*P*=0.0031		
**Fig. 2D**	miR-124 level between WT and KO (n=9 for each group)	Yes	two-sided Student's *t*-test			
	MiR-124-1 HPC	Yes		*P=0.5661*	t =0.654	
	MiR-124-1 BC	Yes		*P=0.3985*	t=0.46	
	MiR-124-2 HPC	Yes		*P=0. 6632*	t=0.383	
	MiR-124-2 BC	Yes		*P=0.7752*	t =0.9108	
	MiR-124-3 HPC	Yes		*P <0.0001*	t=22.059	
	MiR-124-3 BC	Yes		*P <0.0001*	t=34.438	
	MiR-124 HPC	Yes		*P <0.0001*	t=19.66	
	MiR-124 BC	Yes		*P <0.0001*	t=21.851	
**Fig. 2E**	mRNA level of Bhlhe23 and the Ythdf1 between WT and KO (n=4 for each group)	Yes	two-sided Student's *t*-test			
	Bhlhe23	Yes		*P=0.2247*	t=1.294	
	Pri-miR-124-3	Yes		*P <0.0001*	t=20.11	
	Ythdf1	Yes		*P=0.7808*	t =0.29	
**Fig. 3B**	Time savings for different ITIs between WT and KO (n=16 for each group)	Yes	two-sided Student's *t*-test			
	5sec	Yes		*P=0.0011*	t=3.364	
	20min	Yes		*P* <0.001	t=4.898	
	2h	Yes		*P*=0.0011	t=3.377	
	4h	Yes		*P*=0.0041	t=2.946	
	Length savings for different ITIs between WT and KO (n=16 for each group)	Yes	two-sided Student's *t*-test			
	5sec	Yes		*P*=0.0016	t=3.265	
	20min	Yes		*P* <0.001	t=4.591	
	2h	Yes		*P*=0.0003	t=3.765	
	4h	Yes		*P*=0.0016	t=3.266	
**Fig. 3D**	Time to find the hidden platform between WT and KO (n=11 for each group)	Yes	two-way ANOVA followed by Tukey *post hoc* test			Time: $F_{(3, 21)}$ = 39.53, *P<0.0001;* Group: $F_{(1, 7)}$ = 23.82, *P=0.0018;* Interaction: $F_{(3, 21)}$ = 0.9221, *P=0.4473.*
**Fig. 3E**	*left panel*	Yes	two-sided Student's *t*-test	*P=0.018*	t=2.99	
	medial panel	Yes	two-sided Student's *t*-test	*P=0.0435*	t=2.219	
	right panel	Yes	two-sided Student's *t*-test	*P=0.002*	t=3.787	
**Fig. 3F**	Total time	Yes	two-sided Student's *t*-test	*P*=0.6478	t=0.4642	
	Preference for novel object	Yes	two-sided Student's *t*-test	*P*=0.0037	t=3.328	
**Fig. 4A**	LTP at SC-CA1 synapses in the hippocampus (n = 9 slices from 3 mice for both genotypes)	No	the Mann-Whitney U test	*P<0.0001*		
**Fig. 4B**	LTD at SC-CA1 synapses in the hippocampus (n = 10 slices from 4 mice for both genotypes)	No	the Mann-Whitney U test	*P<0.0001*		
**Fig. 4C**	Spine density of basal and apical dendrites of CA1 neurons (n=5 for each group)	No	the Mann-Whitney U test			
	DG outer layer	No		*P*=0.1449		
	CA1 basal	No		*P=0.0136*		
	CA1 apical	No		*P<0.0001*		
**Fig. 4D**	Protein levels of Syp and Psd95 in hippocampus (n=3)	No	the Mann-Whitney U test			

© 2022. Lui K et al. Published online at http://www.aginganddisease.org/EN/10.14336/AD.2022.0204

SUPPLEMENTARY DATA

	Syn	No		*P*=0.01		
	Psd95	No		P=0.0145		
**Fig. 5C**		Yes	two-tailed Student's t-test (n=6 for each group)			
	Cortex					
	RyR3	Yes		*P*=0.0021		
	Hippocampus					
	RyR3	Yes		*P*=0.00145		
**Fig. 5D (*UPPER*)**	Luciferase assay	Yes	one-way ANOVA followed by Tukey *post hoc* test.	*P*=0.0001		$F_{(3, 8)} = 30.33$
	0nM vs5nM	Yes		*P*>0.9999		
	0nM vs10nM	Yes		*P*=0.007		
	0nM vs15nM	Yes		*P*=0.002		
	RyR3mutant	Yes		*P*=0.6052		$F_{(3, 8)}$=0.6493
	pmirGLO	Yes		*P*=0.6631		$F_{(3, 8)}$= 0.5484
**Fig. 5D (*LOWER*)**	Luciferase assay of miR-124-3 inhibitor	Yes	two-sided Student's t-test			
	RYR3	Yes		*P*=0.0017	t=4.02	
	mutant	Yes		*P*=0.3924	t=0.51	
	pmirGLO	Yes		*P*=0.6896	t=0.32	
**Fig. 6B**	RYR3 protein level	Yes	one-way ANOVA followed by Tukey post hoc test	*P*<0.0001		$F_{(2, 15)} = 166.4$
	WT+vehicle vs KO+vehicle	Yes		*P*<0.0001		
	KO+vehicle vs KO+shRNA	Yes		*P*<0.0001		
**Fig. 6C**	Time to find the hidden platform between WT and KO (n=13, 19 and 16 mice for WT+vehicle, KO+vehicle and KO+ShRyr3 group, respectively)	Yes	two-way ANOVA followed by Tukey *post hoc* test	*P*<0.0001		Grouped: $F_{(2, 225)}$ =33.07
				P<0.0001		Time：$F_{(4, 225)}$ = 61.19
				P=0.4563		Interaction: $F_{(8, 225)}$ = 0.925
**Fig. 6D**	Parameters for assessing acquired memory (n=13, 19 and 16 mice for WT+vehicle, KO+vehicle and KO+ShRyr3 group, respectively)	Yes	one-way ANOVA followed by Tukey *post hoc* test			
	Platform crossing	Yes		*P*=0.0241		$F_{(2, 45)}$=4.054
	Time in the quadrant (%)	Yes		*P*=0.0111		$F_{(2, 45)}$=4.982
	Latency to target	Yes		*P*=0.0216		$F_{(2, 45)}$=4.179
**Fig. 6E**	LTP at SC-CA1 synapses in the hippocampus (n=14 slices from 6 mice per genotypes)	Yes	one-way ANOVA followed by Tukey *post hoc* test	*P*<0.0001		$F_{(2, 90)}$=4674
	Wild-type+Vehicle vs KO+Vehicle	Yes		*P*<0.0001		
	Wild-type+Vehicle vs KO+RyR3	Yes		*P*<0.0001		
	KO+Vehicle vs KO+RyR3	Yes		*P*<0.0001		
**Fig. 6f**	LTD at SC-CA1 synapses in the hippocampus (n=13 slices from 6 mice per genotypes)	Yes	one-way ANOVA followed by Tukey *post hoc* test	*P*<0.0001		$F_{(1.798, 53.95)}$=2832
	Wild-type+Vehicle vs KO+Vehicle	Yes		*P*<0.0001		
	Wild-type+Vehicle vs KO+RyR3	Yes		*P*<0.0001		
	KO+Vehicle vs KO+RyR3	Yes		*P*<0.0001		
**Fig. 6G**	Calcium fluorescence (n= 8 slices from 4 mice per group)	Yes	two-way ANOVA followed by Tukey *post hoc* test	*P*=0.0014		Interaction: $F_{(2, 24)}$ = 8.777
		Yes		*P*=0.0002		Treatment: $F_{(1, 24)}$ = 19.26
		Yes		*P*<0.0001		Group: $F_{(2, 24)}$ = 132.3
	WT+ Vehicle (Before Glutamate) vs. WT+vehicle (After Glutamate)	Yes		*P*<0.0001		
	KO+Vehicle (Before Glutamate) vs KO+Vehicle (After Glutamate)	Yes		*P*=0.9942		
	KO + shRyR3(Before Glutamate) vs KO + shRyR3(After Glutamate)	Yes		*P*=0.0002		

© 2022. Lui K et al. Published online at http://www.aginganddisease.org/EN/10.14336/AD.2022.0204

	WT + Vehicle(Before Glutamate) vs KO + Vehicle(Before Glutamate)	Yes		*P*<0.0001		
	KO+shRyr3(Before Glutamate) vs KO + Vehicle(Before Glutamate)	Yes		*P*=0.003		
	KO + Vehicle(Before Glutamate) vs WT + Vehicle(Before Glutamate)	Yes		*P*<0.0001		
**Fig. 7B**	Apoptotic neuron counting	Yes	two-sided Student's t-test	*P*=0.0021	t=4.755	
**Fig. 7C**	Apoptotic neuron range	Yes	two-sided Student's t-test	*P*<0.0001	t=14.56	

Supplementary Table 3. Chromosomal locations and precursor sequences of different miR-124 family members[1]

	Pre-microRNA sequence	Location
Hsa-Pre-miR-124-1	AGGCCUCUCUCUCCGUGUUCACAGCGGACCUUGA UUUAAAUGUCCAUACAAUUAAGGCACGCGGUGAA UGCCAAGAAUGGGGCUG	chr8(-): 9903388-9903472
Hsa-Pre-miR-124-2	AUCAAGAUUAGAGGCUCUGCUCUCCGUGUUCACA GCGGACCUUGAUUUAAUGUCAUACAAUUAAGGCA CGCGGUGAAUGCCAAGAGCGGAGCCUACGGCUGCA CUUGAA	chr8(+): 64379149-64379257
Hsa-Pre-miR-124-3	UGAGGGCCCCUCUGCGUGUUCACAGCGGACCUUG AUUUAAUGUCUAUACAAUUAAGGCACGCGGUGAA UGCCAAGAGAGGCGCCUCC	chr20(+): 63178500-63178586
Mmu-Pre-miR-124-1	AGGCCUCUCUCUCCGUGUUCACAGCGGACCUUGA UUUAAAUGUCCAUACAAUUAAGGCACGCGGUGAA UGCCAAGAAUGGGGCUG	chr14(+): 64590657-64590741
Mmu-Pre-miR-124-2	AUCAAGAUCAGAGACUCUGCUCUCCGUGUUCACA GCGGACCUUGAUUUAAUGUCAUACAAUUAAGGCA CGCGGUGAAUGCCAAGAGCGGAGCCUACGGCUGCACUUGAA	chr3(+): 17795662-17795770
Mmu-Pre-miR-124-3	CUCUGCGUGUUCACAGCGGACCUUGAUUUAAUGU CUAUACAAUUAAGGCACGCGGUGAAUGCCAAGAG	chr2(+): 180894040-180894107

[1]The sequences underline are mature miR-124 sequence.

Supplementary Table 4. Information of primary antibodies for western blots.

Target	Dilution ratio	Source	Product code	Country
Syp	1:1000	Proteintech	17785-1-AP	China
Psd95	1:1000	Cell Signaling Technology	3409	USA
RyR3	1:1000	MilliporeSigma	AB9082	USA
Slit1	1:1000	ABclonal	A16430	China
Ptbp1	1:1000	ABclonal	A1831	China
Ank3	1:1000	Proteintech	27980-1-AP	China
Notch2	1:1000	ABclonal	A0560	China
App	1:1000	ABclonal	A11019	China
Map3k3	1:1000	ABclonal	A16058	China
P65	1:1000	Proteintech	10745-1-AP	China
Cav-1	1:1000	Proteintech	16447-1-AP	China
beta-actin	1:1000	Proteintech	60008-1-Ig	China
Bax	1:1000	Proteintech	50599-2-Ig	China
Caspase1	1:1000	Abcam	ab1872	USA
GSDMD	1:1000	Abcam	ab209845	USA
p16lnk4a	1:1000	Abcam	ab211542	USA
GAPDH	1:1000	Proteintech	60004-1-Ig	China

© 2022. Lui K et al. Published online at http://www.aginganddisease.org/EN/10.14336/AD.2022.0204
